# Resistance proportions for eight priority antibiotic-bacterium combinations in OECD, EU/EEA and G20 countries 2000 to 2030: a modelling study

**DOI:** 10.2807/1560-7917.ES.2019.24.20.1800445

**Published:** 2019-05-16

**Authors:** Tiago Cravo Oliveira Hashiguchi, Driss Ait Ouakrim, Michael Padget, Alessandro Cassini, Michele Cecchini

**Affiliations:** 1 Organisation for Economic Co-operation and Development (OECD), Paris, France; 2 European Centre for Disease Prevention and Control (ECDC), Stockholm, Sweden

**Keywords:** Antimicrobial resistance, Drug resistance, Forecasting, Multilevel analysis, Machine learning, Statistical Distributions, Modelling

## Abstract

**Background:**

Antimicrobial resistance is widely considered an urgent global health issue due to associated mortality and disability, societal and healthcare costs.

**Aim:**

To estimate the past, current and projected future proportion of infections resistant to treatment for eight priority antibiotic-bacterium combinations from 2000 to 2030 for 52 countries.

**Methods:**

We collated data from a variety of sources including ResistanceMap and World Bank. Feature selection algorithms and multiple imputation were used to produce a complete historical dataset. Forecasts were derived from an ensemble of three models: exponential smoothing, linear regression and random forest. The latter two were informed by projections of antibiotic consumption, out-of-pocket medical spending, populations aged 64 years and older and under 15 years and real gross domestic product. We incorporated three types of uncertainty, producing 150 estimates for each country-antibiotic-bacterium-year.

**Results:**

Average resistance proportions across antibiotic-bacterium combinations could grow moderately from 17% to 18% within the Organisation for Economic Co-operation and Development (OECD; growth in 64% of uncertainty sets), from 18% to 19% in the European Union/European Economic Area (EU/EEA; growth in 87% of uncertainty sets) and from 29% to 31% in Group of Twenty (G20) countries (growth in 62% of uncertainty sets) between 2015 and 2030. There is broad heterogeneity in levels and rates of change across countries and antibiotic-bacterium combinations from 2000 to 2030.

**Conclusion:**

If current trends continue, resistance proportions are projected to marginally increase in the coming years. The estimates indicate there is significant heterogeneity in resistance proportions across countries and antibiotic-bacterium combinations.

## Introduction

Antimicrobial resistance (AMR) is a growing global health issue with significant implications for present and future rates of mortality and disability, as well as societal and healthcare costs. According to a comprehensive analysis of the health and economic burden of AMR, an estimated 33,110 patients died in 2015 in European countries from infections due to 16 antibiotic-resistant bacteria [1]. An earlier study found antibiotic resistance resulted in 2.5 million extra hospital days in 2007, an additional EUR 900 million in hospital costs and EUR 1.5 billion in societal costs (including productivity losses from longer stays in hospital and premature mortality) [2]. In the United States (US) in 2013, an estimated 23,000 people died every year from infections with antibiotic-resistant bacteria [3].

In 2014, the Prime Minister of the United Kingdom (UK) commissioned the ‘Review on Antimicrobial Resistance’, which estimated that globally around 700,000 people die each year as a result of resistant infections due to *Staphylococcus aureus*, *Escherichia coli*, *Klebsiella pneumoniae*, malaria, HIV and tuberculosis [4]. The Review projected that drug resistance could claim the lives of 10 million people yearly around the world by 2050. According to the World Bank, the projected economic impact could represent 3.8% of global gross domestic product (GDP) and lead to an additional 1.2 trillion US dollar (USD; approximately EUR 1.0 trillion in 2018) in annual healthcare expenditures and 28.3 million more people living in extreme poverty [5].

These figures have been used extensively by the media, experts and international organisations to illustrate the serious implications of a post-antibiotic world and convey the urgent need for action. However, the assumptions behind these estimates have been far less publicised and have drawn criticism [6]; one critique has been that the projections are not empirically derived but rather based on scenarios. The main scenario sees resistance proportions increase by 40% in all countries in the first year of the analysis, then stay constant until 2050 alongside a doubling of infection rates [6]. 

In this study, we use an ensemble model of three statistical methods to take advantage of available national-level data on drug resistance and its correlates. This model aims to derive estimates of the resistance proportions for eight priority antibiotic-bacterium combinations from 2000 to 2030 for 52 countries (including countries in the European Union/European Economic Area (EU/EEA), the Group of Twenty (G20) and the Organisation for Economic Co-operation and Development (OECD), including key partners and accession countries). Combining the predictions of models with different assumptions can be advantageous when there is uncertainty as to which model or specification best captures the underlying phenomena. Given that a sample of models that have been randomly selected can often outperform the best individual model [7], ensembles are increasingly used to forecast metrics in health [8,9]. Based on the predictions, which are empirically driven and incorporate various types of uncertainty, we discuss previous forecasting scenarios and identify priority areas, not only for data collection and aggregation, but also for policy actions.

## Methods

### Overview

We estimated resistance proportions (share of infections that are resistant to drug treatment) for eight priority antibiotic-bacterium combinations (third-generation cephalosporin-resistant *E. coli*, fluoroquinolone-resistant *E. coli*, penicillin-resistant *Streptococcus pneumoniae*, metcillin-resistant *S. aureus* (MRSA), carbapenem-resistant *K. pneumoniae*, third-generation cephalosporin-resistant *K. pneumoniae*, carbapenem-resistant *Pseudomonas aeruginosa* and vancomycin-resistant *Enterococcus facealis* and *E. faecium*) for each year from 2000 through to 2030 for 52 countries (Argentina, Australia, Austria, Belgium, Brazil, Bulgaria, Canada, Chile, China, Colombia, Costa Rica, Croatia, Cyprus, the Czech Republic, Denmark, Estonia, Finland, France, Germany, Greece, Hungary, Iceland, India, Indonesia, Ireland, Israel, Italy, Japan, Korea, Latvia, Lithuania, Luxembourg, Malta, Mexico, the Netherlands, New Zealand, Norway, Peru, Poland, Portugal, Romania, Russia, Saudi Arabia, Slovakia, Slovenia, South Africa, Spain, Sweden, Switzerland, Turkey, the United Kingdom and the United States) including all OECD, EU/EEA and G20 countries, based on past trends of the relationships between resistance proportions, economic and demographic growth as well as healthcare utilisation. Information on which member states make up the OECD, EU/EEA and G20, can be found in the supplementary material. Some countries belong to more than one group.

Antibiotic-bacterium pairs were selected based on: (i) significance of the burden of disease in the OECD and the EU/EEA, both in the healthcare sector and the community, (ii) policy priority for OECD, G20 and EU/EEA countries [10], (iii) availability of data on resistance and correlates of resistance and, (iv) inclusion in the World Health Organisation (WHO) global priority list of antibiotic-resistant bacteria to guide research and development of new antibiotics [11].

**Figure 1:**
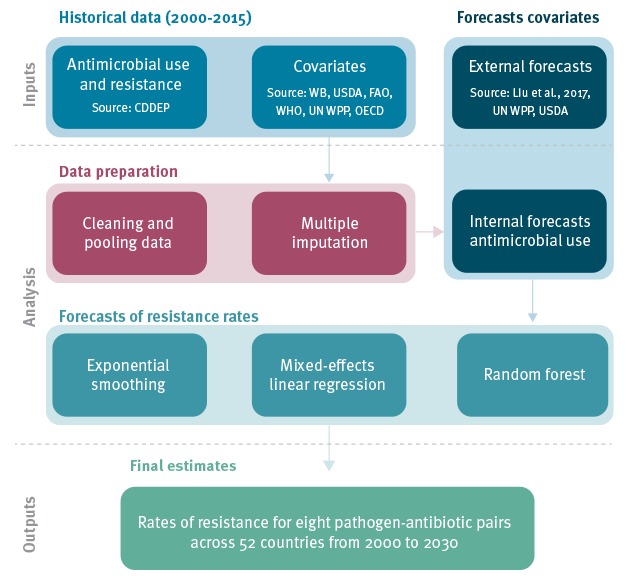
Process diagram for estimating historical and future resistance proportions for eight antibiotic-bacterium combinations in 52 OECD, EU/EEA and G20 countries, 2000–2030

CDDEP: Center for Disease Dynamics, Economics and Prevention; EU/EEA: European Union/European Economic Area; FAO: Food and Agriculture Organization; G20: Group of Twenty; OECD: Organisation for Economic Co-operation and Development; UN WPP: United Nations World Population Prospects; USDA: United States Department of Agriculture; WHO: World Health Organization.

Figure 1 outlines the processes used to estimate future resistance proportions. When we refer to specific geographical regions (e.g. Eastern Europe), we refer to those used by the Statistics Division of the United Nations.

All analyses and estimations were completed using R, version 3.4.1 (R Foundation for Statistical Computing, Vienna, Austria).

### Input data

We extracted data on yearly resistance proportions and antibiotic consumption from public and private national and international surveillance networks, aggregated under ResistanceMap, a web-based set of visualisations with harmonised data [12]. We complemented data from ResistanceMap with nationally representative data from surveillance reports (as detailed in the supplementary material).

A large proportion of the observations were missing (ca 11% on consumption and ca 53% on resistance). To derive a complete dataset, we collated surveillance data with statistics on economic and socio-demographic indicators selected based on previously hypothesised relationships with antibiotic consumption and resistance [13-16], as well as tentative associations posited by the authors and collected from databases of the OECD, the World Bank and specialised agencies and programmes of the United Nations (e.g. the Food and Agriculture Organisation). We used feature selection algorithms to narrow down the initial list of indicators. Indicators with near-zero variance, indicators missing more than 10 percent of observations and indicators that were highly correlated with other indicators were excluded, leaving only the one indicator with least correlation with other variables in the set (see supplementary material for complete and narrowed list of indicators). Building on the narrowed down list of potential correlates, the multiple imputation algorithm Amelia II was then used to fill in missing values for antibiotic consumption and resistance [17].

To inform forecasts of resistance proportions, we looked for projections of the economic and socio-demographic indicators used to impute missing values. We were able to find projections up to 2030 for private per capita household out-of-pocket spending on medical care [18], populations aged over 64 and under 15 [19] and real GDP [20]. We were unable to find projections of antibiotic consumption, so we projected consumption as described in the following section. Forecasts for other potentially relevant covariates (e.g. trade, agricultural production, sanitation, etc.) could not be externally sourced and were not included. Data for all countries, antibiotic-bacterium pairs and years were pooled to maximise statistical power. A more detailed discussion of all analytical work is provided in the supplementary material.

### Estimating future antibiotic consumption and resistance

We used an ensemble model to forecast resistance proportions from 2016 to 2030 drawing on historical trends and relationships with covariates between 2000 and 2015. Resistance proportions were defined as the shares of infections of a given bacterium that were resistant to treatment with a given antibiotic. Three classes of models were used to capture past trends and both linear and nonlinear relationships with covariates: exponential smoothing with an additive damped trend, mixed-effects linear regression and random forest. While mechanistic models of AMR emergence and spread could be a valuable addition to our ensemble [21,22], we did not find any that could inform forecasts at the national level for different countries and priority antibiotic-bacterium combinations [23] and therefore decided against using this approach. To forecast antibiotic consumption – defined as the number of standard units (the equivalent of one pill, capsule or ampoule) per 1,000 population – for each antibiotic class, we used country-specific exponential smoothing with an additive damped trend.

The first model, exponential smoothing, is a popular forecasting tool especially when coupled with a damped trend, a parameter that constrains the trend to a flat line at some point in the future [24]. The forecasted resistance proportion for a future year is the weighted average of the resistance proportions in previous years, with weights decaying exponentially as the observations go further into the past (i.e. when predicting the resistance proportion in 2020, the proportion in 2010 will be more important than the proportion in 2000). The forecasts were computed for each country using smoothing parameters for levels and trends, as well as a damping parameter. The values for these parameters were derived through optimisation with the objective of minimising the sum of squared errors.

The second model, mixed-effects linear regression, was used to maximise statistical power from pooling the data while allowing the relationship between antibiotic resistance and consumption to differ between countries and, within countries between bacteria. The base specification included a random intercept and a random slope on antibiotic consumption. To the base specification we added different combinations of independent variables logged and lagged up to 5 years: (i) real GDP (in 2010 USD), (ii) private per capita household out-of-pocket spending on medical care (in purchasing power parity 2011 USD) and, (iii) population aged 65 years and over and population aged under the age of 15 years. Altogether 2,401 different specifications were tested.

Specifications that did not meet the following inclusion criteria were dropped: all independent variables were statistically significant (α = 0.1), the coefficient on the antibiotic consumption fixed effect was positive and the coefficients on population variables (both aged 65 years and over and under 15 years) were positive. The specifications that passed the inclusion criteria were rerun on data for the period 2000–09 and used to predict resistance proportions for 2010–15. Predictions for each country were compared with the actual historical resistance proportions in that country and the root-mean-square error (RMSE) was computed. The specification with the lowest RMSE was selected and used to forecast resistance proportions up to 2030, making use of the complete historical data (between 2000 and 2015) for each country.

The third method, random forests, are collections of many uncorrelated regression trees (we used 2,000) with each individual tree providing estimates that are averaged to produce final forecasts. A regression tree (like a decision tree) consists of multiple nested ‘if-else’ statements which partition the data. For each partition (i.e. at the end of each branch), a resistance proportion is predicted. While random forests are not easily interpretable they have certain advantages over linear regression methods [25]. First, it is not necessary to specify the form of the relationship between the predictors and resistance proportions (as is the case with linear regression). Second, the method implicitly selects the variables that minimise predictive error. For these reasons, there was no need to test different specifications as with linear regression. The same set of independent variables used in the mixed-effects models was used in the random forests.

### Incorporating and propagating uncertainty

Previous forecasts of drug resistance have been criticised for not accounting for uncertainty [6]. In this study, we incorporated and propagated uncertainty in the imputation of missing values, model selection and specification and model parameters. Because a significant share of observations from ResistanceMap were missing, we incorporated uncertainty from multiple imputation in the final projections of resistance by imputing 150 values for each missing observation, resulting in 150 complete historical datasets.

Uncertainty in model selection and specification was incorporated in the following ways. First, in the absence of strong evidence of which model was most appropriate, we used three classes of models to weaken the implicit assumption that the relationships captured by each method in the past will persist in the future. Second, uncertainty in model specification was captured by the use of different linear regression specifications for each country.

Uncertainty in model parameters was captured by drawing new parameter estimates from a multivariate normal distribution of the estimated linear regression fixed effects (e.g. antibiotic consumption, real GDP, population aged over 64 years, etc.) as the mean and the model’s variance-covariance as the variance. Because the random forest is a stochastic process, a different seed was used each time to ensure that some uncertainty in the model’s parameters was also captured. Uncertainty in the parameters of the exponential smoothing method was not incorporated.

The three types of uncertainty were incorporated in cascade. First, we imputed 150 complete historical datasets capturing uncertainty in the imputation of missing values. Then, we randomly selected without replacement and assigned 50 datasets to each of the three forecasting methods used, thus incorporating uncertainty in model selection and model specification. Thirdly, we incorporated uncertainty in model parameters for the mixed-effects models and the random forests. This process resulted in 150 unique estimates for each year of resistance proportion forecasts (we limited uncertainty datasets to 150 for computational reasons). We report point estimates, lower and upper uncertainty intervals based on the mean and 2.5th and 97.5th percentiles of the 150 sets of forecasts.

## Results

**Figure 2:**
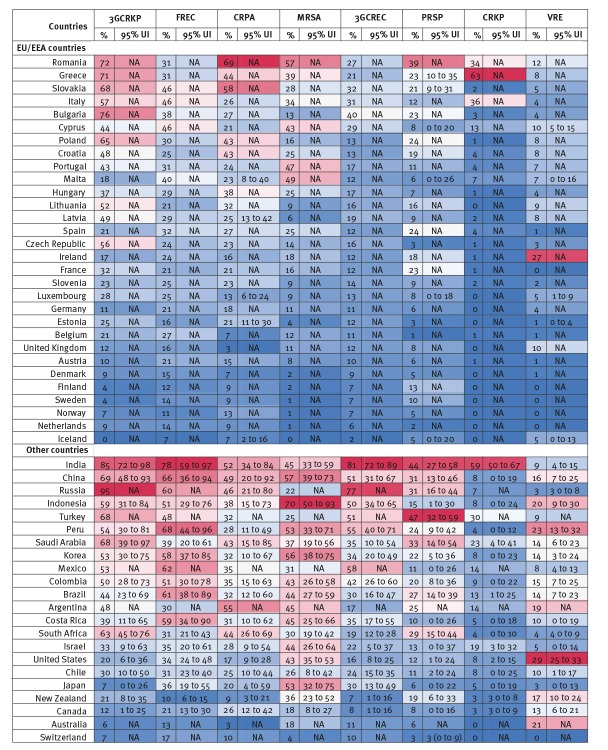
Resistance proportions for eight priority antibiotic-bacterium combinations by country, 52 OECD, EU/EEA and G20 countries, 2015

Figure 2 presents estimates of the resistance proportions for eight priority antibiotic-bacterium combinations in 2015. The proportions of infections from eight bacteria that were resistant to specific antibiotic treatments were lowest in Iceland, the Netherlands and Norway, with simple average proportions of around 5%. Proportions were highest in India, China, Russia and Romania, where two infections in five were estimated to be resistant to treatment. For some priority antibiotic-bacterium combinations, only one infection in five was from susceptible bacteria, a resistance proportion of 80%. There was considerable heterogeneity in average resistance proportions across countries. Within the EU/EEA, the highest resistance proportions were eight times higher than the lowest resistance proportions.

EU/EEA: European Union/European Economic Area; CRKP: carbapenem-resistant *Klebsiella pneumoniae*; CRPA: carbapenem-resistant *Pseudomonas aeruginosa*; FREC: fluoroquinolone-resistant *Escherichia coli*; 3GCREC: third-generation cephalosporin-resistant *Escherichia coli*; 3GCRKP: third-generation cephalosporin-resistant *K. pneumoniae;* G20: Group of Twenty; MRSA: meticillin-resistant *Staphylococcus aureus*; NA: not applicable; OECD: Organisation for Economic Co-operation and Development; PRSP: penicillin-resistant *Streptococcus pneumoniae*; UI: uncertainty intervals; VRE: vancomycin-resistant *Enterococcus faecalis* and *E. faecium*.

95% uncertainty intervals are from multiple imputation; NA indicates there is no uncertainty from multiple imputation.

Order of countries by average resistance proportion across all antibiotic-bacterium combinations; antibiotic-bacterium combinations are sorted from left to right in descending resistance.

Information on which member states make up the OECD, EU/EEA and G20 countries can be found in the supplementary material. Some countries can belong to more than one group (25 EU/EEA countries are also members of the OECD and four EU/EEA countries are also members of the G20, plus the EU as a whole).

The colour scheme is column-specific and based on a 256 point scale (minimum in blue, median in white, maximum in red, points in between coloured proportionally).

Average resistance proportions across all eight priority antibiotic-bacterium combinations were 17% within the OECD, 18% in the EU/EEA and 29% in the G20 countries in 2015. Within the EU/EEA, countries in eastern and southern Europe generally had higher resistance proportions, compared with countries in northern and western Europe.

### Evolution of resistance proportions, 2005–2015

**Figure 3:**
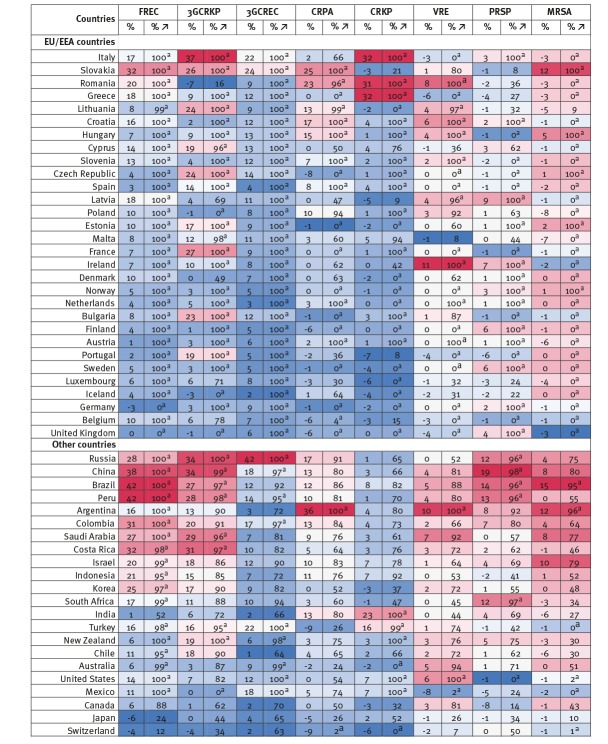
Percentage point changes in resistance proportions for eight priority antibiotic-bacterium combinations by country, 52 OECD, EU/EEA and G20 countries, 2005–2015

Figure 3 presents estimates of the percentage point changes in resistance proportions for eight priority antibiotic-bacterium combinations between 2005 and 2015 (the mean of all 150 uncertainty estimates), as well as the percentage of all 150 uncertainty estimates in which resistance proportions increased; a value of 100 means resistance proportions increased in all 150 uncertainty sets while a value of zero means resistance proportions decreased in all 150 uncertainty sets. The percentages in parentheses give an indication of how likely (or probable) it is that resistance proportions for a certain antibiotic-bacterium combination in a certain country increased between 2005 and 2015, given uncertainty in the underlying data and statistical methods.

EU/EEA: European Union/European Economic Area; CRKP: carbapenem-resistant *Klebsiella pneumoniae*; CRPA: carbapenem-resistant *Pseudomonas aeruginosa*; FREC: fluoroquinolone-resistant *Escherichia coli*; 3GCREC: third-generation cephalosporin-resistant *Escherichia coli*; 3GCRKP: third-generation cephalosporin-resistant *K. pneumonia*; G20: Group of Twenty; MRSA: meticillin-resistant *Staphylococcus aureus*; OECD: Organisation for Economic Co-operation and Development; PRSP: penicillin-resistant *Streptococcus pneumoniae*; VRE: vancomycin-resistant *Enterococcus faecalis* and *E. faecium*.

^a^ There was an increase or decrease in more than 95% of the sets.

The percentage of all 150 uncertainty estimates in which resistance proportions increased between 2005 and 2015 are shown in column ‘% ↗’.

Order of countries according to average resistance proportion across all antibiotic-bacterium combinations, antibiotic-bacterium combinations are sorted from left to right in descending resistance.

Information on which member states make up the OECD, EU/EEA and G20 countries can be found in the supplementary material. Some countries can belong to more than one group (25 EU/EEA countries are also members of the OECD and four EU/EEA countries are also members of the G20, plus the EU as a whole).

The colour scheme is column-specific and based on a 256 point scale (minimum in blue, median in white, maximum in red, points in between coloured proportionally).

On average across all priority antibiotic-bacterium combinations, resistance proportions decreased between 2005 and 2015 in seven countries (Switzerland, the UK, Japan, Belgium, Germany, Iceland and Canada), by on average 2.5 percentage points. It is estimated that in most countries average resistance proportions increased, by as much as 13 percentage points in EU/EEA countries (e.g. the Slovakia and Italy), but potentially even more in Brazil, Russia and China, ca 17 percentage points. There is significant heterogeneity in how resistance proportions evolved across countries.

While it is estimated that no country saw resistance proportions decrease for all eight priority antibiotic-bacterium combinations between 2005 and 2015, in eight countries (Brazil, China, Peru, Argentina, Colombia, Saudi Arabia, Israel and Russia) resistance proportions are estimated to have increased for all eight priority antibiotic-bacterium combinations during that period. In the majority of countries, the evolution in proportions was mixed. For example, in the UK, the proportion of *S. pneumoniae* resistant to penicillin and *E. coli* resistant to third-generation cephalosporins both doubled between 2005 and 2015, from 6% and 4% in 2005, respectively, while resistance proportions to carbapenems in *P. aeruginosa* decreased by 67% from 9% in 2005. In Bulgaria, resistance to third-generation cephalosporins in *K. pneumoniae* went up 23 percentage points, a 43% increase compared with 2005, while the proportion of *S. aureus* resistant to meticillin went down 16 percentage points, a 55% decrease compared with 2005.

Changes in resistance proportions between 2005 and 2015 (using 2005 as the base year) were varied across antibiotic-bacterium combinations and countries. Resistance proportions for MRSA showed the largest decrease, by an estimated 11% across all countries, but actually increased by 2% within the EU/EEA, while the proportion of *E. coli* resistant to third-generation cephalosporins is estimated to have increased by 186% between 2005 and 2015. Croatia and Romania had some of the highest growth rates overall, with average resistance proportions across eight priority antibiotic-bacterium combinations 200% higher in 2015 than 10 years before. Belgium and Portugal had the biggest reductions in estimated resistance proportions compared with 2005 resistance proportions, 10% and 2% respectively. Across all country priority antibiotic-bacterium combinations, predicted resistance proportions in 2015 were between 100% lower and 1,200% higher than in 2005.

### Projection of resistance proportions up to 2030

**Figure 4:**
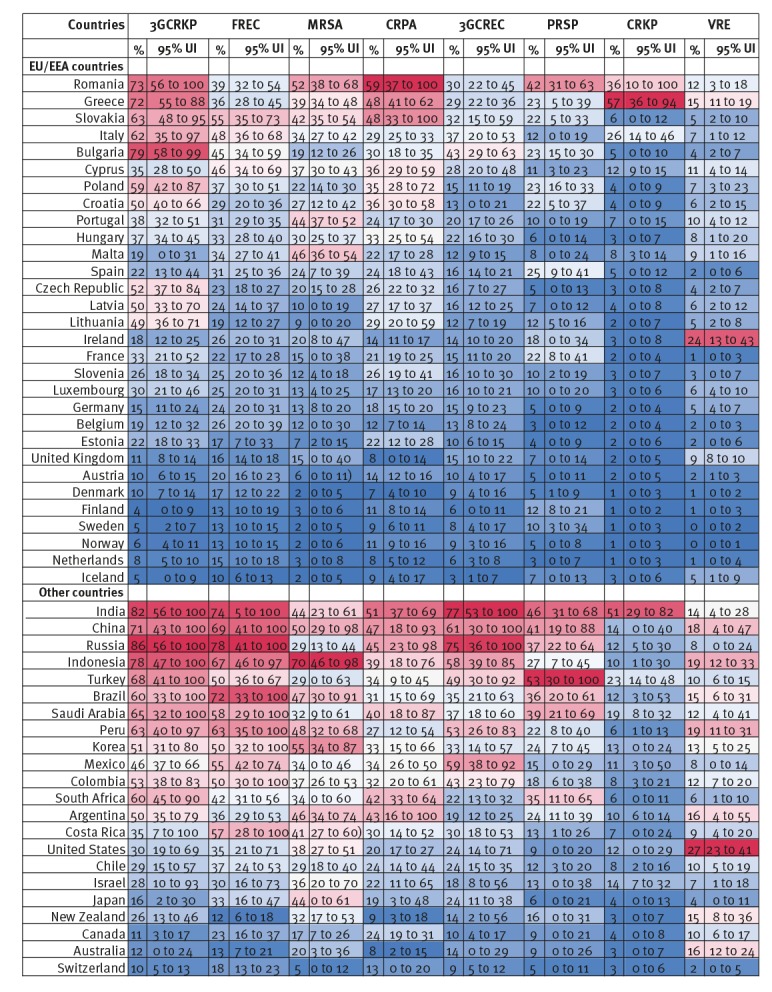
Projected resistance proportions for eight priority antibiotic-bacterium combinations in, 52 OECD, EU/EEA and G20 countries, 2030

Figure 4 presents projections of the resistance proportions in 2030. If past trends and relationships persist in the future, resistance proportions will be slightly higher in 2030 than in 2015 for a majority of country-priority antibiotic-bacterium combinations (64% of combinations), but growth will be slower than for the period between 2005 and 2015, in part due to slower growth in antibiotic-bacterium combinations with higher resistance proportions (e.g. third-generation cephalosporin-resistant *K. pneumoniae*). Of 52 countries for which resistance proportions were projected, the average resistance proportions for eight antibiotic-bacterium combinations could increase in 37 countries and decrease in 13 countries, none of which is estimated to reduce resistance in all eight priority antibiotic-bacterium combinations. In five countries (Bulgaria, Luxembourg, Iceland, Slovenia and Denmark) resistance proportions could increase for all eight priority antibiotic-bacterium combinations. Country-antibiotic-bacterium combinations that are projected to increase are expected to do so four times faster than those that are projected to decrease, a 42% increase compared with a 9% reduction.

EU/EEA: European Union/European Economic Area; CRKP: carbapenem-resistant *Klebsiella pneumoniae*; CRPA: carbapenem-resistant *Pseudomonas aeruginosa*; FREC: fluoroquinolone-resistant *Escherichia coli*; 3GCREC: third-generation cephalosporin-resistant *Escherichia coli*; 3GCRKP: third-generation cephalosporin-resistant *K. pneumonia;* G20: Group of Twenty; MRSA: meticillin-resistant *Staphylococcus aureus*; OECD: Organisation for Economic Co-operation and Development; PRSP: penicillin-resistant *Streptococcus pneumoniae*; UI: uncertainty intervals; VRE: vancomycin-resistant *Enterococcus faecalis* and *E. faecium*.

Order of countries according to average resistance proportion across all antibiotic-bacterium combinations; antibiotic-bacterium combinations are sorted from left to right in descending resistance;

Information on which member states make up the OECD, EU/EEA and G20 countries can be found in the supplementary material. Some countries can belong to more than one group (25 EU/EEA countries are also members of the OECD and four EU/EEA countries are also members of the G20, plus the EU as a whole).

The colour scheme is column-specific and based on a 256 point scale (minimum in blue, median in white, maximum in red, points in between coloured proportionally).

**Figure 5:**
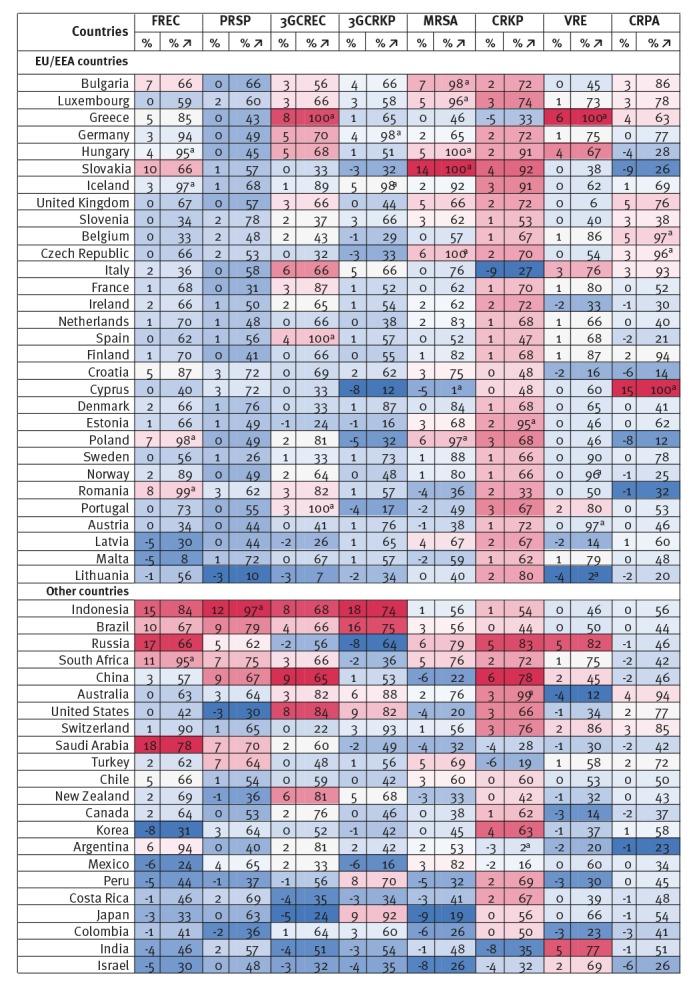
Percentage point changes in resistance proportions for eight priority antibiotic-bacterium combinations, 52 OECD, EU/EEA and G20 countries, 2015–2030

Figure 5 shows the expected mean percentage point change in resistance proportions from 2015 to 2030, as well as the proportion of uncertainty sets where the share of infections with priority resistant bacteria is projected to increase. If the number is higher than 95% then the probability that the resistance proportion will increase in the coming years is considered statistically significant, while if it is lower than 5% then the probability that the resistance proportion will decrease is considered statistically significant.

EU/EEA: European Union/European Economic Area; CRKP: carbapenem-resistant *Klebsiella pneumoniae*; CRPA: carbapenem-resistant *Pseudomonas aeruginosa*; FREC: fluoroquinolone-resistant *Escherichia coli*; 3GCREC: third-generation cephalosporin-resistant *Escherichia coli*; 3GCRKP: third-generation cephalosporin-resistant *K. pneumoniae*, G20: Group of Twenty; MRSA: meticillin-resistant *Staphylococcus aureus*; OECD: Organisation for Economic Co-operation and Development; PRSP: penicillin-resistant *Streptococcus pneumoniae*; VRE: vancomycin-resistant *Enterococcus faecalis* and *E. faecium*.

^a^ There was an increase or decrease in more than 95% of the sets.

The percentage of all 150 uncertainty estimates in which resistance proportions increased between 2015 and 2030 are shown in the column ‘% ↗’.

Order of countries according to average resistance proportion across all antibiotic-bacterium combinations, antibiotic-bacterium combinations are sorted from left to right in descending resistance.

Information on which member states make up the OECD, EU/EEA and G20 countries can be found in the supplementary material. Some countries can belong to more than one group (25 EU/EEA countries are also members of the OECD and four EU/EEA countries are also members of the G20, plus the EU as a whole).

The colour scheme is column-specific and based on a 256 point scale (minimum in blue, median in white, maximum in red, points in between coloured proportionally).

Growth in resistance proportions for third-generation cephalosporin-resistant *E. coli* and *K. pneumoniae*, averaged across all countries, is projected to decrease significantly from 186% and 88% between 2005 and 2015 to 18% and 6% between 2015 and 2030, respectively and with the same pattern across all country groupings. On the other hand, the shares of infections with *E. faecium* and *E. faecalis* resistant to vancomycin are projected to grow at a rate of 63% between 2015 and 2030 in all countries, compared with a growth rate of 12% between 2005 and 2015. Resistance proportions could evolve very differently in different countries and regions. Within the OECD, resistance to penicillin in *S. pneumoniae* could grow faster in the coming years, while the opposite is true within the EU/EEA. Conversely, MRSA in OECD member countries should continue to decline (if more slowly) but is projected to actually increase in EU/EEA countries between 2015 and 2030. Growth in carbapenem resistance among *K. pneumoniae* could intensify in the coming years, across all countries but especially in the G20 countries.

On average across all country-antibiotic-bacterium combinations, resistance proportions could be 23% higher in 2030 compared with 2015, but there is much heterogeneity and uncertainty. In Slovakia, for example, the proportion of *S. aureus* resistant to penicillin could go up by 14 percentage points, from 28% in 2015 to 42% (95% uncertainty interval (UI): 35.84–54.94) in 2030, the largest statistically significant projected increase in the EU/EEA, corresponding to a growth rate of 50%) while the proportion of *P. aeruginosa* resistant to carbapenems is projected to go down in 74% of uncertainty sets over the same period, from 58% to 49% (95% UI: 33.59–100), a growth rate of -16%. In Italy, the share of *K. pneumoniae* resistant to carbapenems is estimated to go down in 73% of uncertainty sets, from 36% to 26% (95% UI: 14.67–46.66), the largest projected reduction within the EU/EEA, a growth rate of -27%, while the proportion of *E. coli* resistant to third-generation cephalosporins could increase by on average six percentage points from 31% to 37% (95% UI: 20.64–53.59), a growth rate of 20%, in 66% of uncertainty sets.

## Discussion

Should current trends in antimicrobial consumption, economic and population growth and health spending continue into the future, resistance proportions are projected to continue to grow in the majority of countries in the OECD, EU/EEA and the G20 in the coming years. We found considerable heterogeneity in levels and rates of change of resistance proportions across countries and priority antibiotic-bacterium combinations, heterogeneity that is missing from forecasting scenarios assuming a single growth rate [4,6].

Consumption of third-generation cephalosporins and fluoroquinolones is projected to increase more slowly than it has in the past years or even decrease (in line with estimates in [26]), in part explaining the expected slowing of the growth of resistance proportions among *E. coli* and *K. pneumoniae*. However, more than a third of *K. pneumoniae* in EU/EEA countries and close to half in G20 countries, were already resistant to third-generation cephalosporins in 2015. As the share of bacteria resistant to second-line treatments keeps growing, the use of third-line treatments like carbapenems could increase, promoting the emergence and spread of carbapenem-resistant bacteria [27]. While carbapenem resistance in *K. pneumoniae* was relatively low in 2015 it is projected to increase, indicating that the need for new antibiotics to treat carbapenem and third-generation cephalosporin-resistant *Enterobacteriaceae* (including *E. coli* and *K. pneumoniae*) is already critical [11].

In EU/EEA and G20 countries, resistance proportions to carpabenems (a third-line treatment) in *K. pneumoniae* is growing faster than resistance to third-generation cephalosporins and fluoroquinolones (second-line treatments) in *E. coli* and *K. pneumoniae*, especially in Europe. If resistance to third-line treatments keeps rising, the remaining therapeutic options may be limited to polymyxins or combinations of antimicrobials, which might carry more side effects to the patient. Even these treatments of last resort might become less effective in the near future, as demonstrated by emerging resistance to polymyxins in Greece and Italy [1].

Patterns of resistance among difficult-to-treat microorganisms like enterococci (e.g. *E. faecalis* and *E. faecium*) and *P. aeruginosa* are also potentially concerning, as these bacteria are intrinsically resistant to several antimicrobial agents and are challenging to contain in healthcare settings [1]. In 2015, almost two in three infections with *P. aeruginosa* involved bacteria resistant to carbapenems in Romania and Slovakia. Although these two countries could see resistance proportions decrease by 2030, in Greece, Turkey and Bulgaria, resistance is expected to keep rising. In 2015, at least 25% of *P. aeruginosa* were already resistant to carbapenems in the latter countries. Though starting from lower 2015 levels, vancomycin resistance in *E. faecalis* and *E. faecium* is projected to increase in a majority of countries.

Most countries have made considerable progress in reducing MRSA in the recent past, but not all. Resistance proportions are estimated to have actually increased in Slovakia, Hungary, Russia and Czech Republic between 2005 and 2015, a trend that is projected to continue up to 2030. In South Africa, Croatia and Bulgaria, reductions in resistance proportions achieved in the past 10 years could be reversed. MRSA remains high in certain countries and the transfer of healthcare-associated clones into the community suggests comprehensive cross-sector policies are needed [27].

The factors behind observed differences in levels and rates of change of resistance proportions across countries and antibiotic-bacterium combinations are difficult to pinpoint with available national-level data. Heterogeneity is likely associated with differences in antimicrobial use, infection prevention and control and the use of healthcare services [27], but also differences in measurement. More broadly, antimicrobial resistance has been linked to a wide range of other factors including animal health and sanitation, agricultural and livestock production, urbanisation and population density, migration and trade, economic growth and governance and population structure [13,14]. Empirically testing the direction and magnitude of these relationships is challenging due to two related factors. First, a lack of comprehensive and comparable data. Many low- and middle-income countries do not have national surveillance systems and even high-income countries have gaps. Second, a limited theoretical understanding of how all these factors are interrelated. The same variable can have both direct and indirect, as well as linear and nonlinear effects.

Multivariate models of antibiotic resistance have limited predictive and explanatory power as illustrated by some of the wide uncertainty intervals reported here. In addition, there are four limitations to this research that should be noted. First, the availability of comparable and reliable data on resistance proportions (and correlates of resistance) across a large number of countries is limited. Internationally comparable data on some indicators likely to be associated with resistance proportions, such as consumption and resistance in the animal sector, could not be found and therefore were not included for a significant number of countries and years. Second, despite efforts from ResistanceMap to harmonise data on resistance and consumption (e.g. by including only invasive isolates from blood and cerebrospinal fluid, categorising results according to CLSI/EUCAST criteria, etc.), many factors could affect the comparability of rates, from differences in frequency of blood culture sampling to coverage and national representativeness of surveillance networks. Third, information on past, current and future policies that might affect resistance proportions have not been explicitly accounted for in the statistical models, although country effects were used in the linear regression. Fourth, achieving predictive accuracy can come at the cost of interpretability. To account for uncertainty in model selection and specification, we combined the results of three classes of models. One of these, random forest, is difficult to interpret because, among other reasons, it aggregates the predictions of thousands of uncorrelated regression trees which allow explanatory variables to interact nonlinearly. Fifth, any forecasting model which relies on past relationships to predict the future is bound by those relationships. Potential future developments that are not possible to account for in the models described here include the more probable (e.g. policies that limit, or otherwise, the use of antibiotics in the human and animal sectors) as well as the more uncertain (e.g. climate change could lead to increased urbanisation, conflict and population displacement, all of which could affect resistance proportions). While the use of three classes of methods limits interpretability to some extent, it also helps minimise the chances of incorrectly projecting historical patterns into the future. Finally, this study estimates resistance proportions, not number of infections. The overall burden of infections from antimicrobial-resistant bacteria is a consequence of both the share of infections from resistant bacteria (estimated here) but also the number of new overall infections, or incidence (not estimated here). These two indicators can evolve differently over time.

Despite the limitations, this study makes use of comprehensive, comparable, international data to forecast resistance proportions for multiple antibiotic-bacterium pairs in a large number of countries, while including multiple types of uncertainty. The estimates are empirically derived (unlike scenario-based forecasts) and while not all sources of uncertainty were accounted for (e.g. uncertainty in forecasts of covariates), the inclusion of uncertainty in the imputation of missing values, model selection and specification and model parameters, is an important step forward (as suggested by de Kraker et al. [6]). Furthermore, as initiatives such as the WHO Global Antimicrobial Resistance Surveillance System (GLASS) and international surveillance networks like European Antimicrobial Resistance Surveillance Network continue to develop, these results can be updated to reflect additional information.

There is a debate around how antimicrobial resistance will evolve in the coming decades [28-30] and what countries should do to prevent a post-antibiotic world. More and better data are essential to produce high-quality estimates that can inform robust policies to address growing drug resistance [31]. It is important not to delay action while surveillance and monitoring systems are being set up. This study provides a set of empirical estimates of potential future rates of antimicrobial resistance to both identify gaps in data and to inform debates around policy actions.

## Supplementary Data

1077000 Supplementary material
