# Supplementary material for: Resistance proportions for eight priority antibiotic-bacterium combinations in OECD, EU/EEA and G20 countries 2000 to 2030: a modelling study
Source: Euro Surveill. 2019 May 16;24(20):1800445. doi: 10.2807/1560-7917.ES.2019.24.20.1800445 (PMC6530255; doi:10.2807/1560-7917.ES.2019.24.20.1800445)
Supplement: Supplementary material [file 18-00445_OLIVEIRA_SupplementaryMaterial.docx]

## Supplementary materials for

## *Resistance proportions for eight priority antibiotic-bacterium combinations in OECD, EU/EEA and G20 countries 2000-2030: a modelling study*

This supplementary material is hosted by Eurosurveillance as supporting information alongside the article *Resistance proportions for eight priority antibiotic-bacterium combinations in OECD, EU/EEA and G20 countries 2000-2030: a modelling study* on behalf of the authors who remain responsible for the accuracy and appropriateness of the content. The same standards for ethics, copyright, attributions and permissions as for the article apply. Eurosurveillance is not responsible for the maintenance of any links or email addresses provided therein.

### Contents

This document contains the following sections:

1. Introduction
2. GATHER statement
3. Geographical scope
4. Input data
5. Multiple imputation of missing values
6. Forecasts of antibiotic consumption and resistance
7. Incorporation of uncertainty
8. Limitations of this study
9. References

### Introduction

Antimicrobial resistance (AMR) is a growing global health threat with significant implications for present and future rates of morbidity and mortality, as well as societal and health care costs. While this assertion is beyond dispute, existing reports of the epidemiological and economic burden of AMR have been criticised as lacking a solid scientific basis [1]. The key challenge is that estimates of the future health and economic burden of AMR must necessarily be preceded by an understanding of how many infections from resistant bacteria there will be in the future. Existing estimates of the future incidence of infections – and the proportion of which are due to bacteria resistant to antimicrobials – have been based on hypothetical scenarios [2], which have been criticised for having no empirical basis and inviting overly confident, potentially erroneous, conclusions [1].

Recent developments in the collection, aggregation and reporting of international surveillance data provide an opportunity to develop a data-driven approach to forecasting resistance proportions, providing a better understanding of the data gaps and uncertainty boundaries. These estimates could then inform policy debates around the impact of what has been done to date, as well as what can be done in the future to contain the emergence and spread of resistant bacteria. This document describes, in detail, the methods used, presents estimates for each country-antibiotic-bacterium combination for a set of years, and assesses the strengths and limitations of this new data-driven approach.


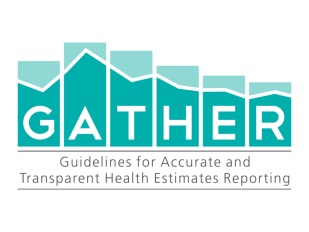


### GATHER statement

We use the Guidelines for Accurate and Transparent Health Estimates Reporting (GATHER) statement to ensure our data and methods are appropriately documented.

| Item # | Checklist item | Reported on section / page # |
| --- | --- | --- |
| Objectives and funding | | |
| 1 | Define the indicator(s), populations (including age, sex, and geographic entities), and time period(s) for which estimates were made. | Reported in the Methods (more specifically in the overview and input data sections). Further information provided in the supplementary materials. |
| 2 | List the funding sources for the work. | Reported in the Acknowledgements. The funder of the study had no role in study design, data collection, data analysis, data interpretation, or writing of the report. |
| Data Inputs | | |
| *For all data inputs from multiple sources that are synthesized as part of the study:* | | |
| 3 | Describe how the data were identified and how the data were accessed. | Reported in the Methods (more specifically in the input data section). |
| 4 | Specify the inclusion and exclusion criteria. Identify all ad-hoc exclusions. | Reported in the Methods (more specifically in the overview section with respect to selection of antibiotic-bacterium pairs, and in the input data section with respect to covariates). |
| 5 | Provide information on all included data sources and their main characteristics. For each data source used, report reference information or contact name/institution, population represented, data collection method, year(s) of data collection, sex and age range, diagnostic criteria or measurement method, and sample size, as relevant. | Reported in the Methods (references to sources in the section input data) and supplementary materials (see Table 1). As relevant, reference information or contact name/institution, population represented, data collection method, year(s) of data collection, sex and age range, diagnostic criteria or measurement method, and sample size, are provided in the data sources (links to data sources provided for each covariate in Table 1 of the supplementary materials, and for other variables in the Methods section). |
| 6 | Identify and describe any categories of input data that have potentially important biases (e.g., based on characteristics listed in item 5). | Reported in the Discussion and in the supplementary materials (more specifically in the sections entitled incorporation of uncertainty and limitations of this study). Specifically, issues related to measurement and reporting of resistance proportions have been highlighted. |
| *For data inputs that contribute to the analysis but were not synthesized as part of the study:* | | |
| 7 | Describe and give sources for any other data inputs. | Reported in the Methods (more specifically in the input data section). Examples of data that contributed to the analyses but were not synthesized include the projections of OOP health spending, GDP per capita and populations over 64 and under 15. |
| *For all data inputs:* | | |
| 8 | Provide all data inputs in a file format from which data can be efficiently extracted (e.g., a spreadsheet rather than a PDF), including all relevant meta-data listed in item 5. For any data inputs that cannot be shared because of ethical or legal reasons, such as third-party ownership, provide a contact name or the name of the institution that retains the right to the data. | Reported in the Methods (more specifically in the input data section) and in the supplementary materials (more specifically in Table 1). While only links to data sources are provided, due to third-party ownership, all data are either freely available or available upon request from respective data sources. |
| Data analysis | | |
| 9 | Provide a conceptual overview of the data analysis method. A diagram may be helpful. | Reported in the Methods (see Figure 1). |
| 10 | Provide a detailed description of all steps of the analysis, including mathematical formulae. This description should cover, as relevant, data cleaning, data pre-processing, data adjustments and weighting of data sources, and mathematical or statistical model(s). | Reported in the Methods (all sections) and in the supplementary materials. |
| 11 | Describe how candidate models were evaluated and how the final model(s) were selected. | Reported in the Methods (more specifically in the sections on forecasting) and in the supplementary materials. |
| 12 | Provide the results of an evaluation of model performance, if done, as well as the results of any relevant sensitivity analysis. | Reported in the supplementary materials (more specifically the section on mixed-effects linear regression). More detailed results (for each country-antibiotic-bacterium-set-specification) are available upon request. No sensitivity analysis was performed. |
| 13 | Describe methods for calculating uncertainty of the estimates. State which sources of uncertainty were, and were not, accounted for in the uncertainty analysis. | Reported in the Methods (more specifically in the section entitled incorporating and propagating uncertainty) and in the supplementary materials (more specifically in the section entitled incorporation of uncertainty). |
| 14 | State how analytic or statistical source code used to generate estimates can be accessed. | Analytic and statistical source code is available from the authors upon request. Note that the code uses parallel computing and requires significant computational resources. |
| Results and Discussion | | |
| 15 | Provide published estimates in a file format from which data can be efficiently extracted. | Estimates in file format available upon request. |
| 16 | Report a quantitative measure of the uncertainty of the estimates (e.g. uncertainty intervals). | Reported in the Results (all sections) and in the supplementary materials. |
| 17 | Interpret results in light of existing evidence. If updating a previous set of estimates, describe the reasons for changes in estimates. | Reported in the Results and Discussion. |
| 18 | Discuss limitations of the estimates. Include a discussion of any modelling assumptions or data limitations that affect interpretation of the estimates. | Reported in the Discussion (paragraphs on limitations) and in the supplementary materials. |

### Geographical scope

Historical and future resistance proportions were estimated for the period between 2000 and 2030 for eight antibiotic-bacterium combinations in 52 countries, including all OECD (including key partners and accession countries), EU/EEA and G20 countries (membership is provided in Table 1 below).

| **Table 1. Country membership of international organisations and regions** | | | | |
| --- | --- | --- | --- | --- |
| **Country** | **OECD** | **OECD Partners/Acccesion** | **EU/EEA** | **G20** |
| Argentina |  |  |  | × |
| Australia | × |  |  | × |
| Austria | × |  | × |  |
| Belgium | × |  | × |  |
| Brazil |  | × |  | × |
| Bulgaria |  |  | × |  |
| Canada | × |  |  | × |
| Chile | × |  |  |  |
| China |  | × |  | × |
| Colombia |  | × |  |  |
| Costa Rica |  | × |  |  |
| Croatia |  |  | × |  |
| Cyprus |  |  | × |  |
| Czech Republic | × |  | × |  |
| Denmark | × |  | × |  |
| Estonia | × |  | × |  |
| Finland | × |  | × |  |
| France | × |  | × | × |
| Germany | × |  | × | × |
| Greece | × |  | × |  |
| Hungary | × |  | × |  |
| Iceland | × |  | × |  |
| India |  | × |  | × |
| Indonesia |  | × |  | × |
| Ireland | × |  | × |  |
| Israel | × |  |  |  |
| Italy | × |  | × | × |
| Japan | × |  |  | × |
| Latvia | × |  | × |  |
| Liechtenstein |  |  | × |  |
| Lithuania | × |  | × |  |
| Luxembourg | × |  | × |  |
| Malta |  |  | × |  |
| Mexico | × |  |  | × |
| The Netherlands | × |  | × |  |
| New Zealand | × |  |  |  |
| Norway | × |  | × |  |
| Peru |  | × |  |  |
| Poland | × |  | × |  |
| Portugal | × |  | × |  |
| Korea | × |  |  | × |
| Romania |  |  | × |  |
| Russia |  |  |  | × |
| Saudi Arabia |  |  |  | × |
| Slovakia | × |  | × |  |
| Slovenia | × |  | × |  |
| South Africa |  | × |  | × |
| Spain | × |  | × |  |
| Sweden | × |  | × |  |
| Switzerland | × |  |  |  |
| Turkey | × |  |  | × |
| United Kingdom | × |  | × | × |
| United States | × |  |  | × |

### Input data

We extracted data on yearly resistance proportions, and antibiotic consumption, from 2000 to 2015 for eight antibiotic-bacterium combinations from national/international sources available from ResistanceMap [3]. We complemented these data with nationally representative data from other surveillance reports, as detailed below.

| **Table 2. Nationally representative data used to complement data from ResistanceMap** | | | | |
| --- | --- | --- | --- | --- |
| **Bacterium** | **Antibiotic** | **Country** | **Year** | **Source** |
| *E. coli* | Cephalosporins (3rd gen) | Japan | 2012 | [4] |
| *E. coli* | Cephalosporins (3rd gen) | China | 2012 | [4] |
| *E. coli* | Cephalosporins (3rd gen) | Australia | 2011 | [4] |
| *E. coli* | Cephalosporins (3rd gen) | Korea | 2012 | [4] |
| *E. coli* | Cephalosporins (3rd gen) | Korea | 2011 | [4] |
| *E. coli* | Fluoroquinolones | China | 2012 | [4] |
| *E. coli* | Fluoroquinolones | Japan | 2012 | [4] |
| *E. coli* | Fluoroquinolones | Korea | 2011 | [4] |
| *E. coli* | Fluoroquinolones | Korea | 2012 | [4] |
| *K. pneumoniae* | Cephalosporins (3rd gen) | China | 2012 | [4] |
| *K. pneumoniae* | Cephalosporins (3rd gen) | Japan | 2012 | [4] |
| *K. pneumoniae* | Cephalosporins (3rd gen) | Korea | 2011 | [4] |
| *K. pneumoniae* | Cephalosporins (3rd gen) | Korea | 2012 | [4] |
| *K. pneumoniae* | Carbapenems | China | 2012 | [4] |
| *K. pneumoniae* | Carbapenems | Japan | 2012 | [4] |
| *K. pneumoniae* | Carbapenems | Korea | 2011 | [4] |
| *K. pneumoniae* | Carbapenems | Korea | 2012 | [4] |
| *S. aureus* | Oxacillin | China | 2012 | [4] |
| *S. aureus* | Oxacillin | Japan | 2012 | [4] |
| *S. aureus* | Oxacillin | Korea | 2011 | [4] |
| *S. aureus* | Oxacillin | Korea | 2012 | [4] |
| *S. pneumoniae* | Penicillins | Australia | 2015 | [5] |
| *S. pneumoniae* | Penicillins | Australia | 2014 | [6] |
| *P. aeruginosa* | Carbapenems | Australia | 2015 | [5] |
| *P. aeruginosa* | Carbapenems | Australia | 2014 | [6] |

Given that 11% of observations on consumption and 53% of observations on resistance proportions were missing, we also collected data on potential correlates of AMR from databases of the OECD, the World Bank, and specialised agencies and programmes of the United Nations (e.g. the Food and Agriculture Organisation), with the objective of imputing missing values taking advantage of all relevant information from observed relationships with covariates. These indicators were selected based on previously hypothesised relationships with antibiotic consumption and resistance [7–10], as well as tentative associations posited by the authors. Table 3 lists all the covariates collected.

| **Table 3. List of potential correlates of AMR for which data was collected** | |
| --- | --- |
| **Description with link to online documentation** | **Source** |
| [Fertilizer consumption (% of fertilizer production)](http://data.worldbank.org/indicator/ag.con.fert.pt.zs) | World Bank |
| [Fertilizer consumption (kilograms per hectare of arable land)](http://data.worldbank.org/indicator/ag.con.fert.zs) | World Bank |
| [Agricultural land (sq. km)](http://data.worldbank.org/indicator/ag.lnd.agri.k2) | World Bank |
| [Agricultural land (% of land area)](http://data.worldbank.org/indicator/ag.lnd.agri.zs) | World Bank |
| [Permanent cropland (% of land area)](http://data.worldbank.org/indicator/ag.lnd.crop.zs) | World Bank |
| [Agricultural irrigated land (% of total agricultural land)](http://data.worldbank.org/indicator/ag.lnd.irig.ag.zs) | World Bank |
| [Average precipitation in depth (mm per year)](http://data.worldbank.org/indicator/ag.lnd.prcp.mm) | World Bank |
| [Food production index (2004-2006 = 100)](http://data.worldbank.org/indicator/ag.prd.food.xd) | World Bank |
| [Livestock production index (2004-2006 = 100)](http://data.worldbank.org/indicator/ag.prd.lvsk.xd) | World Bank |
| [Cereal yield (kg per hectare)](http://data.worldbank.org/indicator/ag.yld.crel.kg) | World Bank |
| [Agriculture, value added (% of GDP)](http://data.worldbank.org/indicator/nv.agr.totl.zs) | World Bank |
| [Improved water source, rural (% of rural population with access)](http://data.worldbank.org/indicator/sh.h2o.safe.ru.zs) | World Bank |
| [Rural poverty headcount ratio at national poverty lines (% of rural population)](http://data.worldbank.org/indicator/si.pov.ruhc) | World Bank |
| [Employment in agriculture (% of total employment)](http://data.worldbank.org/indicator/sl.agr.empl.zs) | World Bank |
| [School enrollment, primary and secondary (gross), gender parity index (GPI)](http://data.worldbank.org/indicator/se.enr.prsc.fm.zs) | World Bank |
| [Prevalence of HIV, total (% of population ages 15-49)](http://data.worldbank.org/indicator/sh.dyn.aids.zs) | World Bank |
| [Mortality rate, under-5 (per 1,000 live births)](http://data.worldbank.org/indicator/sh.dyn.mort) | World Bank |
| [Improved sanitation facilities (% of population with access)](http://data.worldbank.org/indicator/sh.sta.acsn) | World Bank |
| [Incidence of tuberculosis (per 100,000 people)](http://data.worldbank.org/indicator/sh.tbs.incd) | World Bank |
| [Income share held by lowest 20%](http://data.worldbank.org/indicator/si.dst.frst.20) | World Bank |
| [Central government debt, total (% of GDP)](http://data.worldbank.org/indicator/gc.dod.totl.gd.zs) | World Bank |
| [Exports of goods and services (% of GDP)](http://data.worldbank.org/indicator/ne.exp.gnfs.zs) | World Bank |
| [Imports of goods and services (% of GDP)](http://data.worldbank.org/indicator/ne.imp.gnfs.zs) | World Bank |
| [Trade (% of GDP)](http://data.worldbank.org/indicator/ne.trd.gnfs.zs) | World Bank |
| [Chemicals (% of value added in manufacturing)](http://data.worldbank.org/indicator/nv.mnf.chem.zs.un) | World Bank |
| [Adult literacy rate, population 15+ years, both sexes (%)](http://data.worldbank.org/indicator/se.adt.litr.zs) | World Bank |
| [Lower secondary completion rate, total (% of relevant age group)](http://data.worldbank.org/indicator/se.sec.cmpt.lo.zs) | World Bank |
| [Current education expenditure, total (% of total expenditure in public institutions)](http://data.worldbank.org/indicator/se.xpd.ctot.zs) | World Bank |
| [Government expenditure on education, total (% of GDP)](http://data.worldbank.org/indicator/se.xpd.totl.gd.zs) | World Bank |
| [Unemployment, total (% of total labor force) (modeled ILO estimate)](http://data.worldbank.org/indicator/sl.uem.totl.zs) | World Bank |
| [Population ages 0-14 (% of total)](http://data.worldbank.org/indicator/sp.pop.0014.to.zs) | World Bank |
| [Population ages 15-64 (% of total)](http://data.worldbank.org/indicator/sp.pop.1564.to.zs) | World Bank |
| [Access to electricity, rural (% of rural population)](http://data.worldbank.org/indicator/eg.elc.accs.ru.zs) | World Bank |
| [Access to electricity, urban (% of urban population)](http://data.worldbank.org/indicator/eg.elc.accs.ur.zs) | World Bank |
| [Access to non-solid fuel (% of population)](http://data.worldbank.org/indicator/eg.nsf.accs.zs) | World Bank |
| [Fossil fuel energy consumption (% of total)](http://data.worldbank.org/indicator/eg.use.comm.fo.zs) | World Bank |
| [Land area where elevation is below 5 meters (% of total land area)](http://data.worldbank.org/indicator/ag.lnd.el5m.zs) | World Bank |
| [Access to electricity (% of population)](http://data.worldbank.org/indicator/eg.elc.accs.zs) | World Bank |
| [Agricultural methane emissions (thousand metric tons of CO2 equivalent)](http://data.worldbank.org/indicator/en.atm.meth.ag.kt.ce) | World Bank |
| [Agricultural methane emissions (% of total)](http://data.worldbank.org/indicator/en.atm.meth.ag.zs) | World Bank |
| [Droughts, floods, extreme temperatures (% of population, average 1990-2009)](http://data.worldbank.org/indicator/en.clc.mdat.zs) | World Bank |
| [Population living in areas where elevation is below 5 meters (% of total population)](http://data.worldbank.org/indicator/en.pop.el5m.zs) | World Bank |
| [Terrestrial and marine protected areas (% of total territorial area)](http://data.worldbank.org/indicator/er.ptd.totl.zs) | World Bank |
| [Foreign direct investment, net inflows (% of GDP)](http://data.worldbank.org/indicator/bx.klt.dinv.wd.gd.zs) | World Bank |
| [Real effective exchange rate index (2010 = 100)](http://data.worldbank.org/indicator/px.rex.reer) | World Bank |
| [Net migration](http://data.worldbank.org/indicator/sm.pop.netm) | World Bank |
| [International migrant stock, total](http://data.worldbank.org/indicator/sm.pop.totl) | World Bank |
| [International migrant stock (% of population)](http://data.worldbank.org/indicator/sm.pop.totl.zs) | World Bank |
| [Prevalence of anemia among children (% of children under 5)](http://data.worldbank.org/indicator/sh.anm.chld.zs) | World Bank |
| [Prevalence of anemia among non-pregnant women (% of women ages 15-49)](http://data.worldbank.org/indicator/sh.anm.nprg.zs) | World Bank |
| [Condom use, population ages 15-24, female (% of females ages 15-24)](http://data.worldbank.org/indicator/sh.con.1524.fe.zs) | World Bank |
| [Condom use, population ages 15-24, male (% of males ages 15-24)](http://data.worldbank.org/indicator/sh.con.1524.ma.zs) | World Bank |
| [Cause of death, by communicable diseases & maternal, prenatal & nutrition conditions (% of total)](http://data.worldbank.org/indicator/sh.dth.comm.zs) | World Bank |
| [Cause of death, by non-communicable diseases (% of total)](http://data.worldbank.org/indicator/sh.dth.ncom.zs) | World Bank |
| [Women's share of population ages 15+ living with HIV (%)](http://data.worldbank.org/indicator/sh.dyn.aids.fe.zs) | World Bank |
| [Children (0-14) living with HIV](http://data.worldbank.org/indicator/sh.hiv.0014) | World Bank |
| [Prevalence of HIV, female (% ages 15-24)](http://data.worldbank.org/indicator/sh.hiv.1524.fe.zs) | World Bank |
| [Prevalence of HIV, male (% ages 15-24)](http://data.worldbank.org/indicator/sh.hiv.1524.ma.zs) | World Bank |
| [Antiretroviral therapy coverage (% of people living with HIV)](http://data.worldbank.org/indicator/sh.hiv.artc.zs) | World Bank |
| [Immunization, DPT (% of children ages 12-23 months)](http://data.worldbank.org/indicator/sh.imm.idpt) | World Bank |
| [Immunization, measles (% of children ages 12-23 months)](http://data.worldbank.org/indicator/sh.imm.meas) | World Bank |
| [Hospital beds (per 1,000 people)](http://data.worldbank.org/indicator/sh.med.beds.zs) | World Bank |
| [Community health workers (per 1,000 people)](http://data.worldbank.org/indicator/sh.med.cmhw.p3) | World Bank |
| [Nurses and midwives (per 1,000 people)](http://data.worldbank.org/indicator/sh.med.numw.p3) | World Bank |
| [Physicians (per 1,000 people)](http://data.worldbank.org/indicator/sh.med.phys.zs) | World Bank |
| [Use of insecticide-treated bed nets (% of under-5 population)](http://data.worldbank.org/indicator/sh.mlr.nets.zs) | World Bank |
| [Children with fever receiving antimalarial drugs (% of children under age 5 with](http://data.worldbank.org/indicator/sh.mlr.tret.zs) | World Bank |
| [Improved sanitation facilities, rural (% of rural population with access)](http://data.worldbank.org/indicator/sh.sta.acsn.ru) | World Bank |
| [Improved sanitation facilities, urban (% of urban population with access)](http://data.worldbank.org/indicator/sh.sta.acsn.ur) | World Bank |
| [Diabetes prevalence (% of population ages 20 to 79)](http://data.worldbank.org/indicator/sh.sta.diab.zs) | World Bank |
| [Prevalence of underweight, weight for age, female (% of children under 5)](http://data.worldbank.org/indicator/sh.sta.maln.fe.zs) | World Bank |
| [Prevalence of underweight, weight for age, male (% of children under 5)](http://data.worldbank.org/indicator/sh.sta.maln.ma.zs) | World Bank |
| [Prevalence of underweight, weight for age (% of children under 5)](http://data.worldbank.org/indicator/sh.sta.maln.zs) | World Bank |
| [Maternal mortality ratio (modeled estimate, per 100,000 live births)](http://data.worldbank.org/indicator/sh.sta.mmrt) | World Bank |
| [Maternal mortality ratio (national estimate, per 100,000 live births)](http://data.worldbank.org/indicator/sh.sta.mmrt.ne) | World Bank |
| [Diarrhea treatment (% of children under 5 receiving oral rehydration and continued feeding)](http://data.worldbank.org/indicator/sh.sta.orcf.zs) | World Bank |
| [Diarrhea treatment (% of children under 5 who received ORS packet)](http://data.worldbank.org/indicator/sh.sta.orth) | World Bank |
| [Tuberculosis treatment success rate (% of new cases)](http://data.worldbank.org/indicator/sh.tbs.cure.zs) | World Bank |
| [Tuberculosis case detection rate (%, all forms)](http://data.worldbank.org/indicator/sh.tbs.dtec.zs) | World Bank |
| [Newborns protected against tetanus (%)](http://data.worldbank.org/indicator/sh.vac.ttns.zs) | World Bank |
| [External resources for health (% of total expenditure on health)](http://data.worldbank.org/indicator/sh.xpd.extr.zs) | World Bank |
| [Out-of-pocket health expenditure (% of total expenditure on health)](http://data.worldbank.org/indicator/sh.xpd.oopc.to.zs) | World Bank |
| [Health expenditure per capita, PPP (constant 2011 international $)](http://data.worldbank.org/indicator/sh.xpd.pcap.pp.kd) | World Bank |
| [Health expenditure, private (% of GDP)](http://data.worldbank.org/indicator/sh.xpd.priv.zs) | World Bank |
| [Health expenditure, public (% of GDP)](http://data.worldbank.org/indicator/sh.xpd.publ.zs) | World Bank |
| [Health expenditure, total (% of GDP)](http://data.worldbank.org/indicator/sh.xpd.totl.zs) | World Bank |
| [Prevalence of undernourishment (% of population)](http://data.worldbank.org/indicator/sn.itk.defc.zs) | World Bank |
| [Depth of the food deficit (kilocalories per person per day)](http://data.worldbank.org/indicator/sn.itk.dfct) | World Bank |
| [Vitamin A supplementation coverage rate (% of children ages 6-59 months)](http://data.worldbank.org/indicator/sn.itk.vita.zs) | World Bank |
| [Adolescent fertility rate (births per 1,000 women ages 15-19)](http://data.worldbank.org/indicator/sp.ado.tfrt) | World Bank |
| [Mortality rate, adult, female (per 1,000 female adults)](http://data.worldbank.org/indicator/sp.dyn.amrt.fe) | World Bank |
| [Mortality rate, adult, male (per 1,000 male adults)](http://data.worldbank.org/indicator/sp.dyn.amrt.ma) | World Bank |
| [Birth rate, crude (per 1,000 people)](http://data.worldbank.org/indicator/sp.dyn.cbrt.in) | World Bank |
| [Death rate, crude (per 1,000 people)](http://data.worldbank.org/indicator/sp.dyn.cdrt.in) | World Bank |
| [Contraceptive prevalence, any methods (% of women ages 15-49)](http://data.worldbank.org/indicator/sp.dyn.conu.zs) | World Bank |
| [Mortality rate, infant, female (per 1,000 live births)](http://data.worldbank.org/indicator/sp.dyn.imrt.fe.in) | World Bank |
| [Mortality rate, infant (per 1,000 live births)](http://data.worldbank.org/indicator/sp.dyn.imrt.in) | World Bank |
| [Mortality rate, infant, male (per 1,000 live births)](http://data.worldbank.org/indicator/sp.dyn.imrt.ma.in) | World Bank |
| [Life expectancy at birth, total (years)](http://data.worldbank.org/indicator/sp.dyn.le00.in) | World Bank |
| [Fertility rate, total (births per woman)](http://data.worldbank.org/indicator/sp.dyn.tfrt.in) | World Bank |
| [Survival to age 65, female (% of cohort)](http://data.worldbank.org/indicator/sp.dyn.to65.fe.zs) | World Bank |
| [Survival to age 65, male (% of cohort)](http://data.worldbank.org/indicator/sp.dyn.to65.ma.zs) | World Bank |
| [Teenage mothers (% of women ages 15-19 who have had children or are currently pregnant)](http://data.worldbank.org/indicator/sp.mtr.1519.zs) | World Bank |
| [Population ages 65 and above (% of total)](http://data.worldbank.org/indicator/sp.pop.65up.to.zs) | World Bank |
| [Age dependency ratio (% of working-age population)](http://data.worldbank.org/indicator/sp.pop.dpnd) | World Bank |
| [Age dependency ratio, old (% of working-age population)](http://data.worldbank.org/indicator/sp.pop.dpnd.ol) | World Bank |
| [Age dependency ratio, young (% of working-age population)](http://data.worldbank.org/indicator/sp.pop.dpnd.yg) | World Bank |
| [Population growth (annual %)](http://data.worldbank.org/indicator/sp.pop.grow) | World Bank |
| [Unmet need for contraception (% of married women ages 15-49)](http://data.worldbank.org/indicator/sp.uwt.tfrt) | World Bank |
| [Annual freshwater withdrawals, agriculture (% of total freshwater withdrawal)](http://data.worldbank.org/indicator/er.h2o.fwag.zs) | World Bank |
| [Air transport, registered carrier departures worldwide](http://data.worldbank.org/indicator/is.air.dprt) | World Bank |
| [Air transport, freight (million ton-km)](http://data.worldbank.org/indicator/is.air.good.mt.k1) | World Bank |
| [Air transport, passengers carried](http://data.worldbank.org/indicator/is.air.psgr) | World Bank |
| [Railways, goods transported (million ton-km)](http://data.worldbank.org/indicator/is.rrs.good.mt.k6) | World Bank |
| [Railways, passengers carried (million passenger-km)](http://data.worldbank.org/indicator/is.rrs.pasg.km) | World Bank |
| [Rail lines (total route-km)](http://data.worldbank.org/indicator/is.rrs.totl.km) | World Bank |
| [Liner shipping connectivity index (maximum value in 2004 = 100)](http://data.worldbank.org/indicator/is.shp.gcnw.xq) | World Bank |
| [Container port traffic (TEU: 20 foot equivalent units)](http://data.worldbank.org/indicator/is.shp.good.tu) | World Bank |
| [Improved water source, urban (% of urban population with access)](http://data.worldbank.org/indicator/sh.h2o.safe.ur.zs) | World Bank |
| [Improved water source (% of population with access)](http://data.worldbank.org/indicator/sh.h2o.safe.zs) | World Bank |
| [Child employment in agriculture (% of economically active children ages 7-14)](http://data.worldbank.org/indicator/sl.agr.0714.zs) | World Bank |
| [Employment to population ratio, 15+, total (%) (national estimate)](http://data.worldbank.org/indicator/sl.emp.totl.sp.ne.zs) | World Bank |
| [Employment to population ratio, 15+, total (%) (modeled ILO estimate)](http://data.worldbank.org/indicator/sl.emp.totl.sp.zs) | World Bank |
| [Share of youth not in education, employment or training, total (% of youth population)](http://data.worldbank.org/indicator/sl.uem.neet.zs) | World Bank |
| [Adequacy of social protection and labor programs (% of total welfare of beneficiary households)](http://data.worldbank.org/indicator/per_allsp.adq_pop_tot) | World Bank |
| [GINI index (World Bank estimate)](http://data.worldbank.org/indicator/si.pov.gini) | World Bank |
| [Poverty gap at national poverty lines (%)](http://data.worldbank.org/indicator/si.pov.nagp) | World Bank |
| [Poverty headcount ratio at national poverty lines (% of population)](http://data.worldbank.org/indicator/si.pov.nahc) | World Bank |
| [Urban poverty gap at national poverty lines (%)](http://data.worldbank.org/indicator/si.pov.urgp) | World Bank |
| [Urban poverty headcount ratio at national poverty lines (% of urban population)](http://data.worldbank.org/indicator/si.pov.urhc) | World Bank |
| [Ease of doing business index (1=most business-friendly regulations)](http://data.worldbank.org/indicator/ic.bus.ease.xq) | World Bank |
| [Average time to clear exports through customs (days)](http://data.worldbank.org/indicator/ic.cus.durs.ex) | World Bank |
| [Time to import (days)](http://data.worldbank.org/indicator/ic.imp.durs) | World Bank |
| [Logistics performance index: Overall (1=low to 5=high)](http://data.worldbank.org/indicator/lp.lpi.ovrl.xq) | World Bank |
| [International tourism, number of arrivals](http://data.worldbank.org/indicator/st.int.arvl) | World Bank |
| [International tourism, number of departures](http://data.worldbank.org/indicator/st.int.dprt) | World Bank |
| [International tourism, receipts (% of total exports)](http://data.worldbank.org/indicator/st.int.rcpt.xp.zs) | World Bank |
| [Merchandise trade (% of GDP)](http://data.worldbank.org/indicator/tg.val.totl.gd.zs) | World Bank |
| [Food imports (% of merchandise imports)](http://data.worldbank.org/indicator/tm.val.food.zs.un) | World Bank |
| [Food exports (% of merchandise exports)](http://data.worldbank.org/indicator/tx.val.food.zs.un) | World Bank |
| [CPIA quality of public administration rating (1=low to 6=high)](http://data.worldbank.org/indicator/iq.cpa.padm.xq) | World Bank |
| [CPIA trade rating (1=low to 6=high)](http://data.worldbank.org/indicator/iq.cpa.trad.xq) | World Bank |
| [CPIA transparency, accountability, and corruption in the public sector rating (1=low to 6=high)](http://data.worldbank.org/indicator/iq.cpa.tran.xq) | World Bank |
| [Research and development expenditure (% of GDP)](http://data.worldbank.org/indicator/gb.xpd.rsdv.gd.zs) | World Bank |
| [Scientific and technical journal articles](http://data.worldbank.org/indicator/ip.jrn.artc.sc) | World Bank |
| [Researchers in R&D (per million people)](http://data.worldbank.org/indicator/sp.pop.scie.rd.p6) | World Bank |
| [High-technology exports (% of manufactured exports)](http://data.worldbank.org/indicator/tx.val.tech.mf.zs) | World Bank |
| [Urban population (% of total)](http://data.worldbank.org/indicator/sp.urb.totl.in.zs) | World Bank |
| [Literacy rate, youth (ages 15-24), gender parity index (GPI)](http://data.worldbank.org/indicator/se.adt.1524.lt.fm.zs) | World Bank |
| [Smoking prevalence, females (% of adults)](http://data.worldbank.org/indicator/sh.prv.smok.fe) | World Bank |
| [Smoking prevalence, males (% of adults)](http://data.worldbank.org/indicator/sh.prv.smok.ma) | World Bank |
| [GDP per capita, PPP (constant 2011 international $)](http://data.worldbank.org/indicator/ny.gdp.pcap.pp.kd) | World Bank |
| [Total calories supply and total protein supply](http://stats.oecd.org/wbos/fileview2.aspx?IDFile=963e6787-5202-4e83-953c-8597e8932c24) | OECD.Stat |
| [Curative (acute) care beds in hospitals, per 1,000 inhabitants](http://stats.oecd.org/wbos/fileview2.aspx?IDFile=261f59d6-0bbe-4c86-ae5c-0dccd5f59f3e) | OECD.Stat |
| [Hospitals, number](http://stats.oecd.org/wbos/fileview2.aspx?IDFile=74208f2a-5431-4d15-bbf7-50bb917944eb) | OECD.Stat |
| [Immunisations against hepatitis B, % children under 1 year old](http://stats.oecd.org/wbos/fileview2.aspx?IDFile=ce2edb22-bac4-4aad-abe8-26670f7f5f37) | OECD.Stat |
| [Immunisation against influenza among the population aged 65 and over](http://stats.oecd.org/wbos/fileview2.aspx?IDFile=304bf86f-dc96-4a99-9030-1c17cc472ebe) | OECD.Stat |
| [Doctors' consultations, total per capita](http://stats.oecd.org/wbos/fileview2.aspx?IDFile=587d7574-6ed6-4408-9c32-481d322936e6) | OECD.Stat |
| [Hospital discharges, infectious and parasitic diseases, per 100,000 pop](http://stats.oecd.org/wbos/fileview2.aspx?IDFile=1aac08d9-99d5-4b52-9aa8-bbc8da33454b) | OECD.Stat |
| [Hospital discharges, tuberculosis, per 100,000 pop](http://stats.oecd.org/wbos/fileview2.aspx?IDFile=1aac08d9-99d5-4b52-9aa8-bbc8da33454b) | OECD.Stat |
| [Hospital discharges, septicemia, per 100,000 pop](http://stats.oecd.org/wbos/fileview2.aspx?IDFile=1aac08d9-99d5-4b52-9aa8-bbc8da33454b) | OECD.Stat |
| [Hospital average length of stay, infectious and parasitic diseases, per 100,000 pop](http://stats.oecd.org/wbos/fileview2.aspx?IDFile=05c5f7a0-a813-4cc1-8a83-0d77343b5b9e) | OECD.Stat |
| [Hospital average length of stay, tuberculosis, per 100,000 pop](http://stats.oecd.org/wbos/fileview2.aspx?IDFile=05c5f7a0-a813-4cc1-8a83-0d77343b5b9e) | OECD.Stat |
| [Hospital average length of stay, septicemia, per 100,000 pop](http://stats.oecd.org/wbos/fileview2.aspx?IDFile=05c5f7a0-a813-4cc1-8a83-0d77343b5b9e) | OECD.Stat |
| [Hospital discharges, per 100,000 pop](http://stats.oecd.org/wbos/fileview2.aspx?IDFile=1aac08d9-99d5-4b52-9aa8-bbc8da33454b) | OECD.Stat |
| [Hospital average length of stay, per 100,000 pop](http://stats.oecd.org/wbos/fileview2.aspx?IDFile=05c5f7a0-a813-4cc1-8a83-0d77343b5b9e) | OECD.Stat |
| [Beds in residential long-term care facilities, per 1,000 persons aged 65 and over](http://stats.oecd.org/wbos/fileview2.aspx?IDFile=4439d7fd-6fd3-4baa-ad56-35245def07f0) | OECD.Stat |
| [Beds in residential long-term care facilities, per 1,000 persons](http://stats.oecd.org/wbos/fileview2.aspx?IDFile=4439d7fd-6fd3-4baa-ad56-35245def07f0) | OECD.Stat |
| [LTC recipients at home, % of population](http://stats.oecd.org/wbos/fileview2.aspx?IDFile=4b1884af-0b8b-4f3f-994a-7cc4b30d73a0) | OECD.Stat |
| [LTC recipients in institutions (other than hospitals), % of population](http://stats.oecd.org/wbos/fileview2.aspx?IDFile=be9656b8-7f61-4a03-a1fc-bc503f459749) | OECD.Stat |
| [Access to improved water sources (%)](http://www.fao.org/faostat/en/#data/FS/metadata) | FAO |
| [Access to improved sanitation facilities (%)](http://www.fao.org/faostat/en/#data/FS/metadata) | FAO |
| [Road density (per 100 square km of land area)](http://www.fao.org/faostat/en/#data/FS/metadata) | FAO |
| [Rail-lines density (per 100 square km of land area)](http://www.fao.org/faostat/en/#data/FS/metadata) | FAO |
| [Political stability and absence of violence/terrorism (index)](http://www.fao.org/faostat/en/#data/FS/metadata) | FAO |
| [Per capita food supply variability (kcal/capita/day)](http://www.fao.org/faostat/en/#data/FS/metadata) | FAO |
| [Livestock (cattle, buffaloes, pigs, sheep, goats, poultry) total per ha of agricultural area](http://www.fao.org/faostat/en/#data/EK/metadata) | FAO |
| [Meat, Export quantity](http://www.fao.org/faostat/en/#data/TP/metadata) | FAO |
| [Meat, Import quantity](http://www.fao.org/faostat/en/#data/TP/metadata) | FAO |
| [Meat production, Total](http://www.fao.org/faostat/en/#data/QL/metadata) | FAO |
| [Milk production, Total](http://www.fao.org/faostat/en/#data/QL/metadata) | FAO |
| [Total population, WHO World Population Prospects](https://esa.un.org/unpd/wpp/Download/Other/Documentation/) | UN WPP |
| [Population density, WHO World Population Prospects](https://esa.un.org/unpd/wpp/Download/Other/Documentation/) | UN WPP |
| [Population aged 0-14, WHO World Population Prospects](https://esa.un.org/unpd/wpp/Download/Other/Documentation/) | UN WPP |
| [Population aged 15-64, WHO World Population Prospects](https://esa.un.org/unpd/wpp/Download/Other/Documentation/) | UN WPP |
| [Population aged 65+, WHO World Population Prospects](https://esa.un.org/unpd/wpp/Download/Other/Documentation/) | UN WPP |

To inform forecasts of resistance proportions, we collected projections up to 2030 for private per capita household out-of-pocket spending on medical care [11], populations aged over 64 and under 15 [12], and real GDP [13]. Forecasts were also informed by antibiotic consumption which was projected using exponential smoothing (more details below).

### Multiple imputation of missing values

The number (and percentage of total) of missing observations/years per country, for each antibiotic-bacterium pair (resistance) and antibiotic class (consumption) is provided in Tables 4 and 5 below. An asterisk next to a country means that country has more than 50% of all years across bacteria and/or antibiotics missing.

| **Table 4. Missingness by country, for each antibiotic-bacterium pair (number and percent)** | | | | | | | | |
| --- | --- | --- | --- | --- | --- | --- | --- | --- |
| **Country** | **3GCREC** | **FREC** | **VRE** | **CRKP** | **3GCRKP** | **MRSA** | **CRPA** | **PRSP** |
| Argentina* | 14 (88%) | 14 (88%) | 15 (94%) | 14 (88%) | 14 (88%) | 14 (88%) | 14 (88%) | 14 (88%) |
| Australia* | 13 (81%) | 13 (81%) | 13 (81%) | 13 (81%) | 13 (81%) | 13 (81%) | 16 (100%) | 16 (100%) |
| Austria | 1 (6%) | 1 (6%) | 2 (13%) | 5 (31%) | 5 (31%) | 0 (0%) | 5 (31%) | 5 (31%) |
| Belgium | 1 (6%) | 1 (6%) | 3 (19%) | 9 (56%) | 9 (56%) | 0 (0%) | 9 (56%) | 5 (31%) |
| Brazil* | 16 (100%) | 16 (100%) | 16 (100%) | 16 (100%) | 16 (100%) | 16 (100%) | 16 (100%) | 16 (100%) |
| Bulgaria | 1 (6%) | 1 (6%) | 10 (63%) | 6 (38%) | 6 (38%) | 0 (0%) | 8 (50%) | 11 (69%) |
| Canada* | 13 (81%) | 13 (81%) | 15 (94%) | 13 (81%) | 13 (81%) | 13 (81%) | 13 (81%) | 13 (81%) |
| Chile* | 15 (94%) | 15 (94%) | 16 (100%) | 16 (100%) | 16 (100%) | 16 (100%) | 16 (100%) | 16 (100%) |
| China* | 15 (94%) | 15 (94%) | 16 (100%) | 15 (94%) | 15 (94%) | 15 (94%) | 16 (100%) | 16 (100%) |
| Colombia* | 16 (100%) | 16 (100%) | 16 (100%) | 16 (100%) | 16 (100%) | 16 (100%) | 16 (100%) | 16 (100%) |
| Costa Rica* | 16 (100%) | 16 (100%) | 16 (100%) | 16 (100%) | 16 (100%) | 16 (100%) | 16 (100%) | 16 (100%) |
| Croatia | 2 (13%) | 2 (13%) | 4 (25%) | 6 (38%) | 6 (38%) | 2 (13%) | 6 (38%) | 6 (38%) |
| Cyprus | 4 (25%) | 4 (25%) | 14 (88%) | 7 (44%) | 7 (44%) | 4 (25%) | 6 (38%) | 16 (100%) |
| Czech Republic | 1 (6%) | 1 (6%) | 1 (6%) | 6 (38%) | 5 (31%) | 0 (0%) | 5 (31%) | 5 (31%) |
| Denmark | 5 (31%) | 5 (31%) | 6 (38%) | 6 (38%) | 6 (38%) | 0 (0%) | 7 (44%) | 5 (31%) |
| Estonia | 1 (6%) | 1 (6%) | 9 (56%) | 7 (44%) | 5 (31%) | 1 (6%) | 9 (56%) | 5 (31%) |
| Finland | 1 (6%) | 1 (6%) | 1 (6%) | 5 (31%) | 5 (31%) | 0 (0%) | 5 (31%) | 5 (31%) |
| France | 2 (13%) | 2 (13%) | 2 (13%) | 5 (31%) | 5 (31%) | 1 (6%) | 5 (31%) | 5 (31%) |
| Germany | 0 (0%) | 0 (0%) | 1 (6%) | 5 (31%) | 5 (31%) | 0 (0%) | 5 (31%) | 5 (31%) |
| Greece | 0 (0%) | 0 (0%) | 0 (0%) | 5 (31%) | 5 (31%) | 0 (0%) | 5 (31%) | 16 (100%) |
| Hungary | 1 (6%) | 1 (6%) | 3 (19%) | 5 (31%) | 5 (31%) | 1 (6%) | 5 (31%) | 5 (31%) |
| Iceland* | 1 (6%) | 1 (6%) | 16 (100%) | 16 (100%) | 14 (88%) | 0 (0%) | 16 (100%) | 9 (56%) |
| India* | 9 (56%) | 9 (56%) | 13 (81%) | 9 (56%) | 9 (56%) | 10 (63%) | 10 (63%) | 16 (100%) |
| Indonesia* | 16 (100%) | 16 (100%) | 16 (100%) | 16 (100%) | 16 (100%) | 16 (100%) | 16 (100%) | 16 (100%) |
| Ireland | 2 (13%) | 2 (13%) | 2 (13%) | 6 (38%) | 5 (31%) | 0 (0%) | 6 (38%) | 5 (31%) |
| Israel* | 16 (100%) | 16 (100%) | 16 (100%) | 16 (100%) | 16 (100%) | 16 (100%) | 16 (100%) | 16 (100%) |
| Italy | 2 (13%) | 2 (13%) | 1 (6%) | 6 (38%) | 5 (31%) | 0 (0%) | 6 (38%) | 5 (31%) |
| Japan* | 15 (94%) | 15 (94%) | 16 (100%) | 15 (94%) | 15 (94%) | 15 (94%) | 16 (100%) | 16 (100%) |
| Latvia* | 6 (38%) | 6 (38%) | 15 (94%) | 8 (50%) | 8 (50%) | 4 (25%) | 16 (100%) | 6 (38%) |
| Lithuania | 6 (38%) | 6 (38%) | 13 (81%) | 7 (44%) | 6 (38%) | 6 (38%) | 11 (69%) | 6 (38%) |
| Luxembourg | 1 (6%) | 1 (6%) | 15 (94%) | 10 (63%) | 8 (50%) | 0 (0%) | 10 (63%) | 7 (44%) |
| Malta | 1 (6%) | 1 (6%) | 16 (100%) | 7 (44%) | 7 (44%) | 0 (0%) | 7 (44%) | 15 (94%) |
| Mexico* | 6 (38%) | 5 (31%) | 13 (81%) | 6 (38%) | 8 (50%) | 5 (31%) | 6 (38%) | 16 (100%) |
| The Netherlands | 0 (0%) | 0 (0%) | 1 (6%) | 5 (31%) | 5 (31%) | 0 (0%) | 5 (31%) | 5 (31%) |
| New Zealand* | 3 (19%) | 3 (19%) | 16 (100%) | 5 (31%) | 5 (31%) | 16 (100%) | 16 (100%) | 16 (100%) |
| Norway | 0 (0%) | 0 (0%) | 3 (19%) | 5 (31%) | 5 (31%) | 0 (0%) | 5 (31%) | 5 (31%) |
| Peru* | 16 (100%) | 16 (100%) | 16 (100%) | 16 (100%) | 16 (100%) | 16 (100%) | 16 (100%) | 16 (100%) |
| Poland | 1 (6%) | 1 (6%) | 9 (56%) | 8 (50%) | 6 (38%) | 1 (6%) | 7 (44%) | 8 (50%) |
| Portugal | 1 (6%) | 1 (6%) | 2 (13%) | 7 (44%) | 6 (38%) | 0 (0%) | 6 (38%) | 5 (31%) |
| Korea* | 14 (88%) | 14 (88%) | 16 (100%) | 14 (88%) | 14 (88%) | 14 (88%) | 16 (100%) | 16 (100%) |
| Romania* | 3 (19%) | 3 (19%) | 12 (75%) | 11 (69%) | 10 (63%) | 2 (13%) | 12 (75%) | 11 (69%) |
| Russia* | 15 (94%) | 15 (94%) | 16 (100%) | 14 (88%) | 14 (88%) | 14 (88%) | 16 (100%) | 16 (100%) |
| Saudi Arabia* | 16 (100%) | 16 (100%) | 16 (100%) | 16 (100%) | 16 (100%) | 16 (100%) | 16 (100%) | 16 (100%) |
| Slovakia* | 6 (38%) | 6 (38%) | 11 (69%) | 11 (69%) | 11 (69%) | 6 (38%) | 11 (69%) | 16 (100%) |
| Slovenia | 1 (6%) | 1 (6%) | 4 (25%) | 5 (31%) | 5 (31%) | 0 (0%) | 5 (31%) | 5 (31%) |
| South Africa* | 12 (75%) | 12 (75%) | 12 (75%) | 12 (75%) | 12 (75%) | 12 (75%) | 12 (75%) | 16 (100%) |
| Spain | 1 (6%) | 1 (6%) | 1 (6%) | 5 (31%) | 5 (31%) | 0 (0%) | 5 (31%) | 5 (31%) |
| Sweden | 2 (13%) | 2 (13%) | 2 (13%) | 8 (50%) | 6 (38%) | 1 (6%) | 6 (38%) | 6 (38%) |
| Switzerland* | 13 (81%) | 13 (81%) | 13 (81%) | 13 (81%) | 13 (81%) | 13 (81%) | 13 (81%) | 15 (94%) |
| Turkey* | 13 (81%) | 13 (81%) | 13 (81%) | 13 (81%) | 13 (81%) | 13 (81%) | 13 (81%) | 15 (94%) |
| United Kingdom | 1 (6%) | 1 (6%) | 5 (31%) | 5 (31%) | 5 (31%) | 0 (0%) | 5 (31%) | 5 (31%) |
| United States | 1 (6%) | 1 (6%) | 1 (6%) | 1 (6%) | 1 (6%) | 1 (6%) | 1 (6%) | 3 (19%) |

Legend: * indicates country is missing more than 50% of observations/years. 3GCREC – third-generation cephalosporin-resistant *E. coli*. FREC – fluoroquinolone-resistant *E. coli*. VRE – vancomycin-resistant *E. faecium* and *E. faecalis*. CRKP – carbapenem-resistant *K. pneumoniae*. 3GCRKP – third-generation cephalosporin-resistant *K. pneumoniae*. MRSA – methicillin-resistant *S. aureus*. CRPA – carbapenem-resistant *P. aeruginosa*. PRSP – penicillin-resistant *S. pneumoniae*.

| **Table 5. Missingness by country, for each antibiotic class (number and percent)** | | | | | | | |
| --- | --- | --- | --- | --- | --- | --- | --- |
| **Country** | **Total** | **BSP** | **NSP** | **3GC** | **F** | **G** | **C** |
| Argentina | 0 (0%) | 0 (0%) | 0 (0%) | 0 (0%) | 0 (0%) | 0 (0%) | 0 (0%) |
| Australia | 0 (0%) | 0 (0%) | 0 (0%) | 0 (0%) | 0 (0%) | 0 (0%) | 0 (0%) |
| Austria | 0 (0%) | 0 (0%) | 0 (0%) | 0 (0%) | 0 (0%) | 0 (0%) | 0 (0%) |
| Belgium | 0 (0%) | 0 (0%) | 0 (0%) | 0 (0%) | 0 (0%) | 0 (0%) | 0 (0%) |
| Brazil | 0 (0%) | 0 (0%) | 0 (0%) | 0 (0%) | 0 (0%) | 0 (0%) | 0 (0%) |
| Bulgaria | 0 (0%) | 0 (0%) | 0 (0%) | 0 (0%) | 0 (0%) | 0 (0%) | 0 (0%) |
| Canada | 0 (0%) | 0 (0%) | 0 (0%) | 0 (0%) | 0 (0%) | 0 (0%) | 0 (0%) |
| Chile | 0 (0%) | 0 (0%) | 0 (0%) | 0 (0%) | 0 (0%) | 0 (0%) | 0 (0%) |
| China | 0 (0%) | 0 (0%) | 0 (0%) | 0 (0%) | 0 (0%) | 0 (0%) | 0 (0%) |
| Colombia | 0 (0%) | 0 (0%) | 0 (0%) | 0 (0%) | 0 (0%) | 0 (0%) | 0 (0%) |
| Costa Rica* | 16 (100%) | 16 (100%) | 16 (100%) | 16 (100%) | 16 (100%) | 16 (100%) | 16 (100%) |
| Croatia | 5 (31%) | 5 (31%) | 5 (31%) | 5 (31%) | 5 (31%) | 5 (31%) | 5 (31%) |
| Cyprus* | 16 (100%) | 16 (100%) | 16 (100%) | 16 (100%) | 16 (100%) | 16 (100%) | 16 (100%) |
| Czech Republic | 0 (0%) | 0 (0%) | 0 (0%) | 0 (0%) | 0 (0%) | 0 (0%) | 0 (0%) |
| Denmark | 0 (0%) | 0 (0%) | 0 (0%) | 0 (0%) | 0 (0%) | 0 (0%) | 0 (0%) |
| Estonia | 0 (0%) | 0 (0%) | 0 (0%) | 0 (0%) | 0 (0%) | 0 (0%) | 0 (0%) |
| Finland | 0 (0%) | 0 (0%) | 0 (0%) | 0 (0%) | 0 (0%) | 0 (0%) | 0 (0%) |
| France | 0 (0%) | 0 (0%) | 0 (0%) | 0 (0%) | 0 (0%) | 0 (0%) | 0 (0%) |
| Germany | 0 (0%) | 0 (0%) | 0 (0%) | 0 (0%) | 0 (0%) | 0 (0%) | 0 (0%) |
| Greece | 0 (0%) | 0 (0%) | 0 (0%) | 0 (0%) | 0 (0%) | 0 (0%) | 0 (0%) |
| Hungary | 0 (0%) | 0 (0%) | 0 (0%) | 0 (0%) | 0 (0%) | 0 (0%) | 0 (0%) |
| Iceland* | 16 (100%) | 16 (100%) | 16 (100%) | 16 (100%) | 16 (100%) | 16 (100%) | 16 (100%) |
| India | 0 (0%) | 0 (0%) | 0 (0%) | 0 (0%) | 0 (0%) | 0 (0%) | 0 (0%) |
| Indonesia | 0 (0%) | 0 (0%) | 0 (0%) | 0 (0%) | 0 (0%) | 0 (0%) | 0 (0%) |
| Ireland | 0 (0%) | 0 (0%) | 0 (0%) | 0 (0%) | 0 (0%) | 0 (0%) | 0 (0%) |
| Israel* | 16 (100%) | 16 (100%) | 16 (100%) | 16 (100%) | 16 (100%) | 16 (100%) | 16 (100%) |
| Italy | 0 (0%) | 0 (0%) | 0 (0%) | 0 (0%) | 0 (0%) | 0 (0%) | 0 (0%) |
| Japan | 0 (0%) | 0 (0%) | 0 (0%) | 0 (0%) | 0 (0%) | 0 (0%) | 0 (0%) |
| Latvia | 0 (0%) | 0 (0%) | 0 (0%) | 0 (0%) | 0 (0%) | 0 (0%) | 0 (0%) |
| Lithuania | 0 (0%) | 0 (0%) | 0 (0%) | 0 (0%) | 0 (0%) | 0 (0%) | 0 (0%) |
| Luxembourg | 0 (0%) | 0 (0%) | 0 (0%) | 0 (0%) | 0 (0%) | 0 (0%) | 0 (0%) |
| Malta* | 16 (100%) | 16 (100%) | 16 (100%) | 16 (100%) | 16 (100%) | 16 (100%) | 16 (100%) |
| Mexico | 0 (0%) | 0 (0%) | 0 (0%) | 0 (0%) | 0 (0%) | 0 (0%) | 0 (0%) |
| The Netherlands | 5 (31%) | 5 (31%) | 5 (31%) | 5 (31%) | 5 (31%) | 5 (31%) | 5 (31%) |
| New Zealand | 0 (0%) | 0 (0%) | 0 (0%) | 0 (0%) | 0 (0%) | 0 (0%) | 0 (0%) |
| Norway | 0 (0%) | 0 (0%) | 0 (0%) | 0 (0%) | 0 (0%) | 0 (0%) | 0 (0%) |
| Peru | 0 (0%) | 0 (0%) | 0 (0%) | 0 (0%) | 0 (0%) | 0 (0%) | 0 (0%) |
| Poland | 0 (0%) | 0 (0%) | 0 (0%) | 0 (0%) | 0 (0%) | 0 (0%) | 0 (0%) |
| Portugal | 0 (0%) | 0 (0%) | 0 (0%) | 0 (0%) | 0 (0%) | 5 (31%) | 0 (0%) |
| Korea | 0 (0%) | 0 (0%) | 0 (0%) | 0 (0%) | 0 (0%) | 0 (0%) | 0 (0%) |
| Romania | 0 (0%) | 0 (0%) | 0 (0%) | 0 (0%) | 0 (0%) | 0 (0%) | 0 (0%) |
| Russia | 0 (0%) | 0 (0%) | 0 (0%) | 0 (0%) | 0 (0%) | 0 (0%) | 0 (0%) |
| Saudi Arabia | 0 (0%) | 0 (0%) | 0 (0%) | 0 (0%) | 0 (0%) | 0 (0%) | 0 (0%) |
| Slovakia | 0 (0%) | 0 (0%) | 0 (0%) | 0 (0%) | 0 (0%) | 0 (0%) | 0 (0%) |
| Slovenia | 0 (0%) | 0 (0%) | 0 (0%) | 0 (0%) | 0 (0%) | 0 (0%) | 0 (0%) |
| South Africa | 0 (0%) | 0 (0%) | 0 (0%) | 0 (0%) | 0 (0%) | 0 (0%) | 0 (0%) |
| Spain | 0 (0%) | 0 (0%) | 0 (0%) | 0 (0%) | 0 (0%) | 0 (0%) | 0 (0%) |
| Sweden | 0 (0%) | 0 (0%) | 0 (0%) | 0 (0%) | 0 (0%) | 0 (0%) | 0 (0%) |
| Switzerland | 0 (0%) | 0 (0%) | 0 (0%) | 0 (0%) | 0 (0%) | 0 (0%) | 0 (0%) |
| Turkey | 0 (0%) | 0 (0%) | 0 (0%) | 0 (0%) | 0 (0%) | 0 (0%) | 0 (0%) |
| United Kingdom | 0 (0%) | 0 (0%) | 0 (0%) | 0 (0%) | 0 (0%) | 0 (0%) | 0 (0%) |
| United States | 0 (0%) | 0 (0%) | 0 (0%) | 0 (0%) | 0 (0%) | 0 (0%) | 0 (0%) |

Legend: * indicates country is missing more than 50% of observations/years. Total – all antibiotic consumption. BSP – broad spectrum penicillin. NSP – narrow spectrum penicillin. 3GC – third-generation cephalosporin. F – fluoroquinolone. G – glycopeptides. C – carbapenem.

In line with previous work dealing with missing values in time-series cross-sectional data [14], multiple imputation was used to derive a complete dataset with previously missing observations *filled in* using all relevant information from observed relationships with covariates. The multiple imputation algorithm Amelia II (written for the statistical software R) was used [15]. This algorithm assumes all variables are jointly distributed according to a multivariate normal distribution and that observations are missing at random. Amelia II imputes multiple values for each missing observation, taking into account prediction uncertainty.

Before running the imputation algorithm, a number of pre-processing steps were performed. In order to achieve a joint distribution of the data as close as possible to a multivariate normal distribution, the data was Box-Cox-transformed.

To reduce the number of potential correlates of resistance and consumption from two hundred to a more useful number, feature selection techniques were employed: variables with near-zero variance were excluded; indicators for which more than 10 percent of observations were missing were excluded; indicators which were highly correlated (pair-wise correlation above 0.7 was used) with other indicators were excluded leaving only the one indicator with least correlation with other variables in the dataset. The list of predictors included after feature selection is available below in Table 6.

| **Table 6. List of potential correlates of AMR included after feature selection** | |
| --- | --- |
| **Description with link to documentation** | **Source** |
| [Cereal yield (kg per hectare)](http://data.worldbank.org/indicator/ag.yld.crel.kg) | World Bank |
| [Agriculture, value added (% of GDP)](http://data.worldbank.org/indicator/nv.agr.totl.zs) | World Bank |
| [Unemployment, total (% of total labor force) (modeled ILO estimate)](http://data.worldbank.org/indicator/sl.uem.totl.zs) | World Bank |
| [Fossil fuel energy consumption (% of total)](http://data.worldbank.org/indicator/eg.use.comm.fo.zs) | World Bank |
| [Foreign direct investment, net inflows (% of GDP)](http://data.worldbank.org/indicator/bx.klt.dinv.wd.gd.zs) | World Bank |
| [Immunization, DPT (% of children ages 12-23 months)](http://data.worldbank.org/indicator/sh.imm.idpt) | World Bank |
| [Tuberculosis case detection rate (%, all forms)](http://data.worldbank.org/indicator/sh.tbs.dtec.zs) | World Bank |
| [Out-of-pocket health expenditure (% of total expenditure on health)](http://data.worldbank.org/indicator/sh.xpd.oopc.to.zs) | World Bank |
| [Health expenditure, private (% of GDP)](http://data.worldbank.org/indicator/sh.xpd.priv.zs) | World Bank |
| [Health expenditure, total (% of GDP)](http://data.worldbank.org/indicator/sh.xpd.totl.zs) | World Bank |
| [Adolescent fertility rate (births per 1,000 women ages 15-19)](http://data.worldbank.org/indicator/sp.ado.tfrt) | World Bank |
| [Survival to age 65, male (% of cohort)](http://data.worldbank.org/indicator/sp.dyn.to65.ma.zs) | World Bank |
| [Age dependency ratio (% of working-age population)](http://data.worldbank.org/indicator/sp.pop.dpnd) | World Bank |
| [Population growth (annual %)](http://data.worldbank.org/indicator/sp.pop.grow) | World Bank |
| [Air transport, freight (million ton-km)](http://data.worldbank.org/indicator/is.air.good.mt.k1) | World Bank |
| [Improved water source, urban (% of urban population with access)](http://data.worldbank.org/indicator/sh.h2o.safe.ur.zs) | World Bank |
| [Employment to population ratio, 15+, total (%) (modeled ILO estimate)](http://data.worldbank.org/indicator/sl.emp.totl.sp.zs) | World Bank |
| [International tourism, number of arrivals](http://data.worldbank.org/indicator/st.int.arvl) | World Bank |
| [Merchandise trade (% of GDP)](http://data.worldbank.org/indicator/tg.val.totl.gd.zs) | World Bank |
| [Food imports (% of merchandise imports)](http://data.worldbank.org/indicator/tm.val.food.zs.un) | World Bank |
| [Food exports (% of merchandise exports)](http://data.worldbank.org/indicator/tx.val.food.zs.un) | World Bank |
| [High-technology exports (% of manufactured exports)](http://data.worldbank.org/indicator/tx.val.tech.mf.zs) | World Bank |
| [Urban population (% of total)](http://data.worldbank.org/indicator/sp.urb.totl.in.zs) | World Bank |
| [Population density, WHO World Population Prospects](https://esa.un.org/unpd/wpp/Download/Other/Documentation/) | UN WPP |

It is important to note that the feature selection techniques employed are not intended to select the variables that are most likely to be related to resistance proportions. All the variables in Table 3 have been selected for their potential relevancy. Rather, the feature selection criteria are used to select the group of indicators that are statistically most informative by, for example, eliminating indicators that have no variance across countries and years, or dropping variables that are highly correlated. The purpose of this exercise is to accurately impute antibiotic consumption and resistance proportions. No policy implications should be derived from this process (i.e. included variables are not more policy-relevant than excluded variables).

The imputation then proceeded in the following way: first, total aggregated antibiotic consumption across all antibiotic classes was imputed 150 times; then, individual antibiotic classes and resistance proportions were imputed once for each of those 150 datasets. This chained two-step procedure allows imputed total antibiotic consumption to inform imputations of individual classes of antibiotics and resistance proportions. Coupled with observational priors, this helps ensure that the sums of imputed classes of antibiotics will never go above the imputed total antibiotic consumption. In both steps, the algorithm was run with the following settings: time, country and region effects; a ridge prior of 1% of the number of observations to improve stability (as suggested in Amelia II documentation); bounds to limit consumption to non-negative values; observational priors on missing observations for countries for which there is data on antibiotic consumption in OECD.Stat (only for the first step; discussed in more detail below); and lags and leads for resistance and consumption.

Data on total antibiotic consumption from ResistanceMap is completely missing for 7 countries (Costa Rica, Croatia, Cyprus, Iceland, Israel, Malta and The Netherlands). The OECD.Stat database contains data for three of those countries (Iceland, Israel and The Netherlands) on total consumption of antibiotics. However, ResistanceMap and OECD.Stat use different metrics to characterise consumption: ResistanceMap reports standard units (defined as the equivalent of one pill, capsule or ampoule) per 1,000 inhabitants; the OECD reports defined daily dosage per 1,000 inhabitants per day. To allow data from OECD to inform multiple imputation of total antibiotic consumption from ResistanceMap, observational priors were calculated using ratios. For each of the three countries in both databases:

1. Ratios between consumptions in the three countries and other countries in OECD data were calculated.
2. Ratios from step 1 were multiplied by consumption data from ResistanceMap.
3. The average and standard deviation across results from previous step were calculated.
4. The average and standard deviation for each year were used as observational priors for the three countries.

It would be interesting to understand how well the multiple imputation algorithm predicts missing values, but because these values are missing it is not possible to compare the imputed values to the missing values. Amelia II does however provide a function called *overimpute* which involves treating non-missing observations as if they were missing, generating multiple imputed values and then comparing these imputed values to the initial non-missing observations. The output of this function is a graph showing the imputations for each observation (with 90% confidence intervals) against the true value of that observation. If the majority of imputed values (including the 90% confidence intervals) fall on top of the diagonal line, then the multiple imputation procedure has been successful in predicting actual observations as if they had been missing [15]. To illustrate, figures 1 and 2 show the results of the *overimpute* function for consumption of third-generation cephalosporins and for resistance proportions for *K. pneumoniae* resistant to third-generation cephalosporins.

**Figure 1. Overimputation of consumption of third-generation cephalosporins**


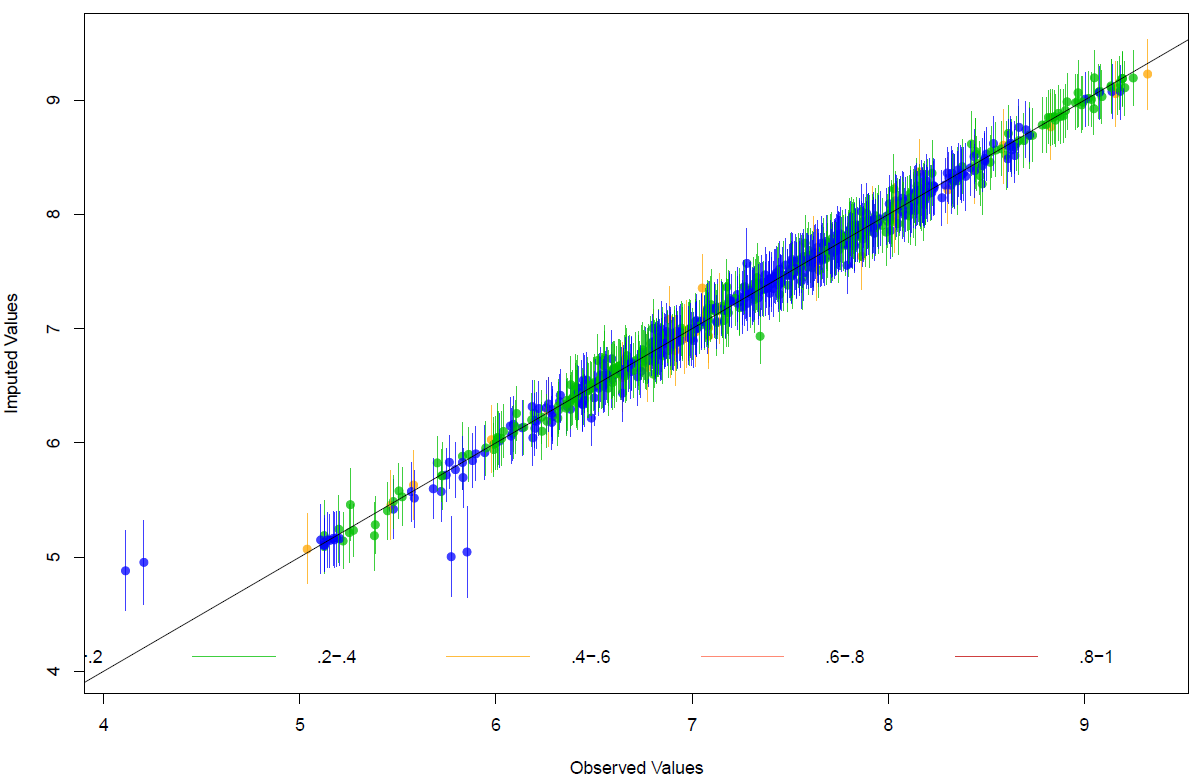


**Figure 2. Overimputation of resistance proportions for *K. pneumoniae* resistant to third-generation cephalosporins**


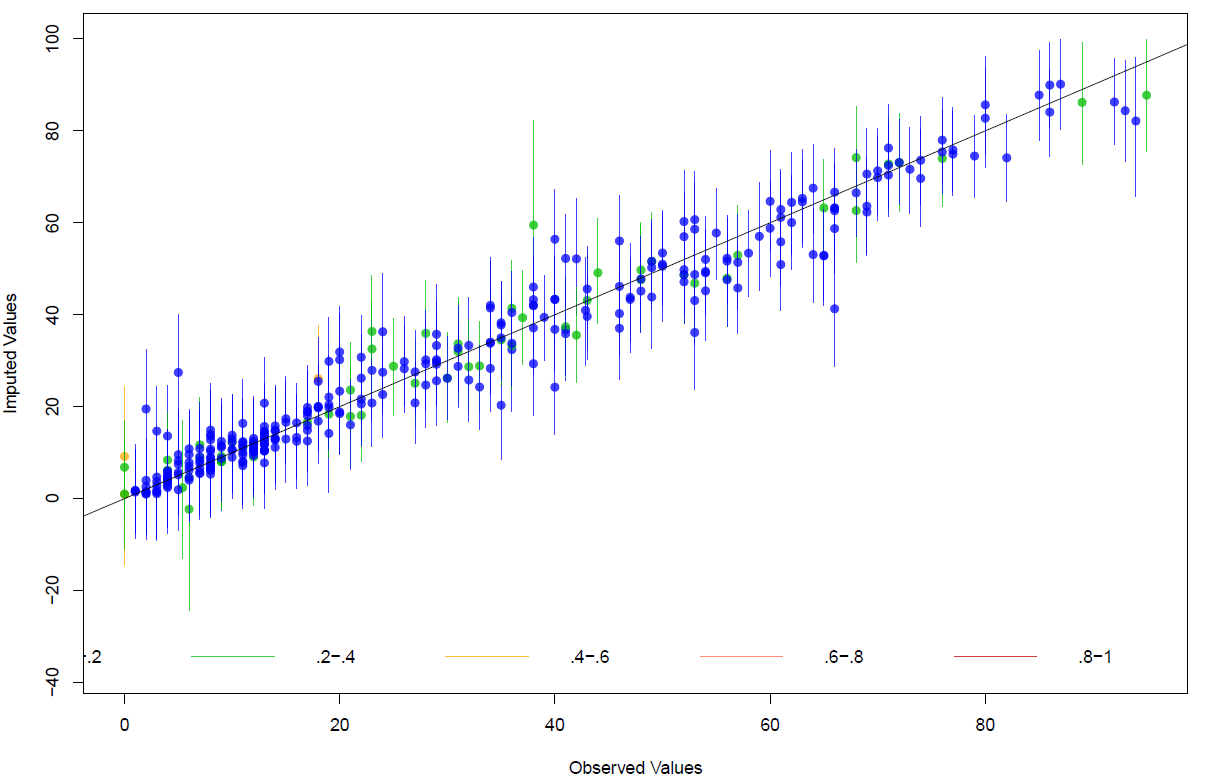


### Forecasts of antibiotic consumption and resistance

Future resistance proportions for the period between 2016 and 2030 were estimated using an ensemble model: a combination of estimates from three classes of models. The three classes of models were chosen to capture different aspects of the underlying phenomena. Each class of models was run on 50 complete datasets, chosen randomly from the 150 imputed datasets. This effectively means the three classes of models are given equal weighting in the final estimates of the ensemble.

The first method, exponential smoothing with an additive damped trend, uses a weighted average of past proportions with more recent observations given a higher weight. The damping parameter ensures projections do not behave unrealistically (e.g., increasing or decreasing exponentially). The second method, a mixed-effects linear regression, was employed to capture the linear relationships between resistance proportions and covariates. The data for all countries, antibiotic-bacterium pairs, and years was pooled to maximise statistical power while allowing the relationship between antibiotic consumption and resistance to differ between countries and, within countries, between bacteria. The third method, random forest, provides an alternative to the linear regression by capturing any non-linear relationships between resistance proportions and covariates. Estimates from random forests are averages of many uncorrelated regression trees (2,000 were used in each imputed dataset). A regression tree – like a decision tree – consists of multiple nested if-else statements which partition the data. For each partition (i.e. at the end of each branch), a resistance proportion is predicted.

While exponential smoothing does not make use of covariates, both linear regression and random forest do. To project resistance proportions using these methods it is then necessary to first project covariates. Forecasts up to 2030 were collected from external sources for the following variables: out-of-pocket (OOP) health expenditure, population by age group and population density, total real and per capita GDP and GDP growth. Given no external source was available, we forecasted antibiotic consumption using exponential smoothing with an additive damped trend on antibiotic use data from ResistanceMap. Forecasts for other variables of potential interest (e.g., trade, agricultural production, sanitation, etc.) could not be externally sourced. In the absence of external data, forecasting these variables in-house was deemed undesirable as the predictive value added would come at the cost of increasing uncertainty.

Random forests are different from linear regressions in many ways but two of those differences are important to note here [16]: the analyst does not need to specify the form of the relationship between the predictors and what is being predicted, and the method implicitly selects the variables that minimize predictive error. Because linear regressions do not share these characteristics with random forests, it is important to test different specifications and select those that minimize predictive error. To do so, different combinations of explanatory variables, in various forms (e.g. log-transformed and different lags), were tested. A total of 2,401 different models were run for each of the 50 imputed datasets randomly assigned to the linear regression. The specifications that met certain prior beliefs on relationships between resistance and covariates, and maximised goodness-of-fit, were selected for each country in each imputed dataset. More details on specification selection are provided below.

This process culminates in 150 complete datasets with historical and future resistance proportions for eight antibiotic-bacterium pairs across 52 countries from 2000 to 2030. Put differently, there are 150 estimates for each country-antibiotic-bacterium-year. These sets of estimates can be described using distributional statistics such as medians, means and percentiles.

As stated, the data for all countries, antibiotic-bacterium pairs, and years were pooled. The first five rows of one of the imputed datasets are shown in the table below to illustrate the data structure:

| **Table 7. First five rows of dataset used in analysis** | | | | | |
| --- | --- | --- | --- | --- | --- |
| **Country** | **Year** | **Bacterium** | **Resistance proportion (%)** | **Antibiotic** | **Consumption** |
| Argentina | 2000 | *K. pneumoniae* | 5.2 | 3GC | 1261 |
| Argentina | 2000 | *E. coli* | 13.2 | 3GC | 1261 |
| Argentina | 2000 | *E. coli* | 10.2 | F | 680 |
| Argentina | 2000 | *E. faecium* & *E. faecalis* | 17.1 | G | 1 |
| Argentina | 2000 | *P. aeruginosa* | 20.5 | C | 2 |

Note: Consumption is defined as the number of standard units (the equivalent of one pill, capsule or ampoule) per 1,000 population; 3GC – third-generation cephalosporin. F – fluoroquinolone. G – glycopeptides. C – carbapenem.

**Model 1: exponential smoothing with additive damped trend**

Exponential smoothing methods are very popular forecasting tools, especially those using a damped trend, a parameter that constrains the trend to a flat line at some point in the future [17]. Hyndman and Athanasopoulos provide a very good discussion of these methods in their 2012 book *Forecasting: principles and practice* [17].

The idea behind exponential smoothing is relatively simple: the forecasted resistance proportion for a future year is the weighted average of the resistance proportions in previous years, with weights decaying exponentially as the observations go further into the past. In other words, when predicting the resistance proportion in 2020, the proportion in 2010 will be more important than the proportion in 2000. We used the following specification:

Forecasting equation ${\hat{\text{y}}}_{\text{t+1|t}}\text{=}\text{l}_{\text{t}}\text{+}\left( \text{ϕ}\text{+}\text{ϕ}^{\text{2}}\text{+}\text{⋯}\text{+}\text{ϕ}^{\text{h}} \right)\text{b}_{\text{t}}$

Smoothing equation (level) $\text{l}_{\text{t}}\text{=}{\text{α}\text{y}}_{\text{t}}\text{+}\left( \text{1-}\text{α} \right)\left( \text{l}_{\text{t-1}}\text{+}{\text{ϕ}\text{b}}_{\text{t-1}} \right)$

Smoothing equation (trend) $\text{b}_{\text{t}}\text{=}\text{β}^{\text{*}}\left( \text{l}_{\text{t}}\text{-}\text{l}_{\text{t-1}} \right)\text{+}\left( \text{1-}\text{β}^{\text{*}} \right)\text{ϕ}\text{b}_{\text{t-1}}$

where $\text{α}$, the smoothing parameter for the level, and $\text{β}^{\text{*}}$ the smoothing parameter for the trend, must both be between 0 and 1, including 0 and 1, $\text{l}_{\text{t}}$ is the level of the series at time *t*, $\text{b}_{\text{t}}$ is the estimate of the trend (slope) of the series at time *t* and $\text{h}$ represents the forecasting step (e.g., starting with historical data up to 2015, $\text{h}$ is equal to 1 when forecasting the value of resistance for 2016, $\text{h}$ is equal to 2 when forecasting the value for 2017, and so on so forth), and finally $\text{ϕ}$ is a damping parameter with value between 0 and 1, not including 0 and 1. When $\text{ϕ}$ is equal to 0, the equations above transform to the case of simple exponential smoothing, while if $\text{ϕ}$ is equal to 1, then the equations simplify to Holt’s linear trend. For other values between 0 and 1, the addition of the damping parameter means that forecasts will converge to

$$\text{l}_{\text{T}}\text{+}\frac{\text{ϕ}\text{b}_{\text{T}}}{\left( \text{1-}\text{ϕ} \right)}$$

as $\text{h→∞}$ (i.e., when forecasting very far into the future). Recall that *T* is the last observation in the series.

While the smoothing parameters $\text{α}$, $\text{β}^{\text{*}}$ and $\text{ϕ}$ may be chosen arbitrarily, a more empirical approach is to derive their values from the data. This can be done through optimisation with initial values and unknown parameters $\text{α}$, $\text{β}^{\text{*}}$ and $\text{ϕ}$ chosen to minimize the sum of the squared errors (SSE):

$$\sum_{\text{t=1}}^{\text{T}} \text{e}_{\text{t}}^{\text{2}}\text{=}\sum_{\text{t=1}}^{\text{T}} \left( \text{y}_{\text{t}}\text{-}{\hat{\text{y}}}_{\text{t|t-1}} \right)^{\text{2}}$$

where $\text{e}_{\text{t}}$ is the error for time *t*: the difference between the observed value $\text{y}_{\text{t}}$ and the estimate ${\hat{\text{y}}}_{\text{t|t-1}}$ for time *t*.

Exponential smoothing with an additive damped trend was used to forecast the resistance proportions for each country-antibiotic-bacterium combination for each of 50 randomly selected imputed datasets, with starting values and unknown parameters $\text{α}$, $\text{β}^{\text{*}}$ and $\text{ϕ}$ chosen to minimise the SSE, so that each country-antibiotic-bacterium-imputed set will have their own set of smoothing parameters. The historical series ran from 2000 to 2015 (parameters T and *t*) and the forecasting horizon was 2016 to 2030 (parameters *h*; equal to 1 for 2016, equal to 2 for 2017, and so on so forth).

**Model 2: mixed-effects linear regressions**

Pooling the data has a positive impact on statistical power; however it is important to correctly specify the relationships between resistance proportions and antibiotic consumption and other explanatory variables. Mixed-effects linear regression models were used to allow the relationship between antibiotic consumption and resistance to differ between countries and, within countries, between bacteria.

The base specification used was:

$$\text{y}_{\text{ij}}\text{=}\text{β}_{\text{0ij}}\text{+}\text{β}_{\text{1ij}}\text{×}\text{C}_{\text{ij}}\text{+}\text{e}_{\text{ij}}\text{=}\left( \text{β}_{\text{0}}\text{+}\text{u}_{\text{0j}}\text{+}\text{u}_{\text{0ij}} \right)\text{+}\left( \text{β}_{\text{1}}\text{+}\text{u}_{\text{1j}}\text{+}\text{u}_{\text{1ij}} \right)\text{×C}_{\text{ij}}\text{+}\text{e}_{\text{ij}}$$

where $\text{y}_{\text{ij}}$ is the resistance proportion for bacterium *i* in country *j*; $\text{β}_{\text{0ij}}$ is a country-specific random intercept which is the sum of country-wide overall mean $\text{β}_{\text{0}}$, country-specific displacement $\text{u}_{\text{0j}}$ and country-bacterium-specific displacement $\text{u}_{\text{0}\text{ij}}$; $\text{β}_{\text{1}\text{i}\text{j}}$ is a country-specific random slope on antibiotic consumption $\text{C}_{\text{ij}}$ of the antibiotic class to which bacterium *i* is resistant in country *j*; $\text{β}_{\text{1}\text{i}\text{j}}$ is the sum of country-wide mean slope $\text{β}_{\text{1}}$, country-specific offset $\text{u}_{\text{1j}}$ and country-bacterium-specific offset $\text{u}_{\text{1ij}}$; and finally, $\text{e}_{\text{ij}}$ is the residual term.

Besides antibiotic consumption, real GDP, OOP health expenditure, population aged 65 and over, and population under the age of 15 were also considered as potential correlates of resistance proportions. Different combinations of these covariates, log-transformed and lagged up to 5 years, were added to the base specification in the following way:

The base specification described above

+ Either none or a single form of total GDP (current or lagged up to 5 years)

+ Either none or a single form of OOP health expenditure (current or lagged up to 5 years)

+ Either none or a single form of population 65+ (current or lagged up to 5 years)

+ Either none or a single form of population 0-14 (current or lagged up to 5 years)

This process results in 2,401 specifications. Examples include:

$$\text{y}_{\text{ij}}\text{=}\text{β}_{\text{0ij}}\text{+}\text{β}_{\text{1ij}}\text{×}\text{C}_{\text{ij}}\text{+}{\text{β}_{\text{2}}\text{×log}\left( \text{GDP}_{\text{ij}} \right)\text{+e}}_{\text{ij}}$$

$$\text{y}_{\text{ij}}\text{=}\text{β}_{\text{0ij}}\text{+}\text{β}_{\text{1ij}}\text{×}\text{C}_{\text{ij}}\text{+}{\text{β}_{\text{3}}\text{×log}\left( \text{Pop65}_{\text{ij}} \right)\text{+ }\text{β}_{\text{5}}\text{×log}\left( \text{OOP}_{\text{ij}}^{\text{lag1}} \right)\text{+e}}_{\text{ij}}$$

$$\text{y}_{\text{ij}}\text{=}\text{β}_{\text{0ij}}\text{+}\text{β}_{\text{1ij}}\text{×}\text{C}_{\text{ij}}\text{+}{\text{β}_{\text{2}}\text{×log}\left( \text{GDP}_{\text{ij}}^{\text{lag2}} \right)\text{+e}}_{\text{ij}}\text{+}{\text{β}_{\text{3}}\text{×log}\left( \text{Pop65}_{\text{ij}}^{\text{lag5}} \right)\text{+}\text{β}_{\text{4}}\text{×log}\left( \text{Pop14}_{\text{ij}} \right)\text{+ }\text{β}_{\text{5}}\text{×log}\left( \text{OOP}_{\text{ij}}^{\text{lag3}} \right)\text{+e}}_{\text{ij}}$$

These specifications are run for each of 50 randomly chosen imputed datasets. Given prior beliefs from the literature, specifications were excluded if: the antibiotic consumption fixed effect was negative or the 95% confidence interval contained zero; the 95% confidence intervals for the coefficients on GDP variables and OOP health expenditure contained zero; the coefficients on population over 64 years old and population under 15 years old were negative or the 95% confidence intervals contained zero. The number of, and percentage of all 2,401 specifications, that passed the inclusion criteria are presented in Table 8 for each of the 50 imputed sets randomly allocated to linear regression.

| **Table 8. Number of (and percentage of total) linear regression specifications that passed the inclusion criteria for each of 50 imputed sets randomly allocated to linear regression** | | |
| --- | --- | --- |
| **Imputed set** | **Specifications that passed inclusion criteria** | **Percentage of all 2,401 specifications** |
| 1 | 163 | 7% |
| 5 | 163 | 7% |
| 6 | 163 | 7% |
| 7 | 163 | 7% |
| 15 | 379 | 16% |
| 22 | 163 | 7% |
| 23 | 343 | 14% |
| 24 | 163 | 7% |
| 25 | 163 | 7% |
| 28 | 163 | 7% |
| 34 | 163 | 7% |
| 42 | 343 | 14% |
| 43 | 163 | 7% |
| 47 | 163 | 7% |
| 49 | 163 | 7% |
| 53 | 163 | 7% |
| 54 | 379 | 16% |
| 59 | 163 | 7% |
| 63 | 163 | 7% |
| 64 | 163 | 7% |
| 67 | 163 | 7% |
| 70 | 163 | 7% |
| 73 | 163 | 7% |
| 80 | 163 | 7% |
| 82 | 379 | 16% |
| 83 | 343 | 14% |
| 87 | 163 | 7% |
| 90 | 163 | 7% |
| 96 | 343 | 14% |
| 100 | 163 | 7% |
| 101 | 163 | 7% |
| 102 | 163 | 7% |
| 103 | 163 | 7% |
| 104 | 163 | 7% |
| 109 | 163 | 7% |
| 113 | 163 | 7% |
| 122 | 163 | 7% |
| 124 | 163 | 7% |
| 125 | 379 | 16% |
| 128 | 163 | 7% |
| 129 | 163 | 7% |
| 130 | 163 | 7% |
| 131 | 251 | 10% |
| 136 | 350 | 15% |
| 137 | 163 | 7% |
| 140 | 163 | 7% |
| 141 | 379 | 16% |
| 144 | 163 | 7% |
| 146 | 163 | 7% |
| 149 | 163 | 7% |

Specifications that passed the exclusion criteria above were tested for goodness-of-fit. The period between 2000 and 2009 was used to train the regression model, and the period between 2010 and 2015 was used to test its predictive power. For each country in each imputed set, the specification that minimizes the RMSE was chosen, resulting in 2,550 best specifications, one for each country-imputed set (all other specifications were excluded). The RMSE for each country, in each imputed dataset, is calculated using the following formula:

$$\sqrt{\frac{\sum_{\text{t=1}}^{\text{n}} \left( {\hat{\text{y}}}_{\text{t}}\text{-}\text{y}_{\text{t}} \right)^{\text{2}}}{\text{n}}}$$

where ${\hat{\text{y}}}_{\text{t}}$ is the predicted resistance proportion and $\text{y}_{\text{t}}$ is the observed resistance proportion, for year t. Table 9 shows the number of unique specifications chosen for each country, and the mean improvement (in percentage) from the worst specification (highest RMSE) to the best specification (lowest RMSE), across all 50 imputed sets.

| **Table 9. Number of unique specifications chosen for each country and average improvement (in percentage) in RMSE from worst specification to best specification across all 50 imputed sets** | | |
| --- | --- | --- |
| **Country** | **# of unique specifications chosen** | **Mean gain in RMSE (%) worst to best specification** |
| Argentina | 1 | 9.9 |
| Australia | 1 | 17.5 |
| Austria | 1 | 17.9 |
| Belgium | 1 | 11.3 |
| Brazil | 16 | 8.4 |
| Bulgaria | 5 | 5.3 |
| Canada | 1 | 15.2 |
| Chile | 1 | 15.3 |
| China | 27 | 6.4 |
| Colombia | 1 | 11.6 |
| Costa Rica | 1 | 11.5 |
| Croatia | 1 | 6.4 |
| Cyprus | 1 | 12.1 |
| Czech Republic | 1 | 10.9 |
| Denmark | 1 | 36.9 |
| Estonia | 1 | 14.0 |
| Finland | 1 | 26.5 |
| France | 1 | 9.3 |
| Germany | 1 | 11.4 |
| Greece | 7 | 7.4 |
| Hungary | 3 | 7.9 |
| Iceland | 1 | 19.6 |
| India | 12 | 2.7 |
| Indonesia | 3 | 3.8 |
| Ireland | 1 | 10.3 |
| Israel | 2 | 9.9 |
| Italy | 8 | 7.8 |
| Japan | 1 | 9.8 |
| Latvia | 2 | 7.2 |
| Lithuania | 1 | 10.7 |
| Luxembourg | 1 | 8.4 |
| Malta | 1 | 11.8 |
| Mexico | 1 | 8.9 |
| The Netherlands | 1 | 39.8 |
| New Zealand | 1 | 13.8 |
| Norway | 1 | 29.5 |
| Peru | 1 | 7.3 |
| Poland | 1 | 9.4 |
| Portugal | 3 | 8.3 |
| Korea | 1 | 9.4 |
| Romania | 8 | 5.1 |
| Russian Fed. | 33 | 4.5 |
| Saudi Arabia | 1 | 5.7 |
| Slovakia | 1 | 6.9 |
| Slovenia | 7 | 10.7 |
| South Africa | 1 | 4.3 |
| Spain | 1 | 9.4 |
| Sweden | 1 | 20.4 |
| Switzerland | 1 | 15.2 |
| Turkey | 1 | 2.9 |
| United Kingdom | 1 | 7.1 |
| United States | 1 | 10.7 |

Note: mean gain (%) = (RMSE of worst specification – RMSE of best specification) / RMSE of worst specification

**Model 3: random forests**

Random forests are collections of many uncorrelated regression trees (2,000 were used in each run) with each individual tree providing estimates which are averaged to produce final forecasts. A regression tree – like a decision tree – consists of multiple nested if-else statements which partition the data. For each partition (i.e., at the end of each branch), a resistance proportion is predicted. A very simple, and merely illustrative, regression tree is provided below (this tree was not used in the analysis, it is merely an example):

**Figure 3. Illustrative example of a regression tree.**


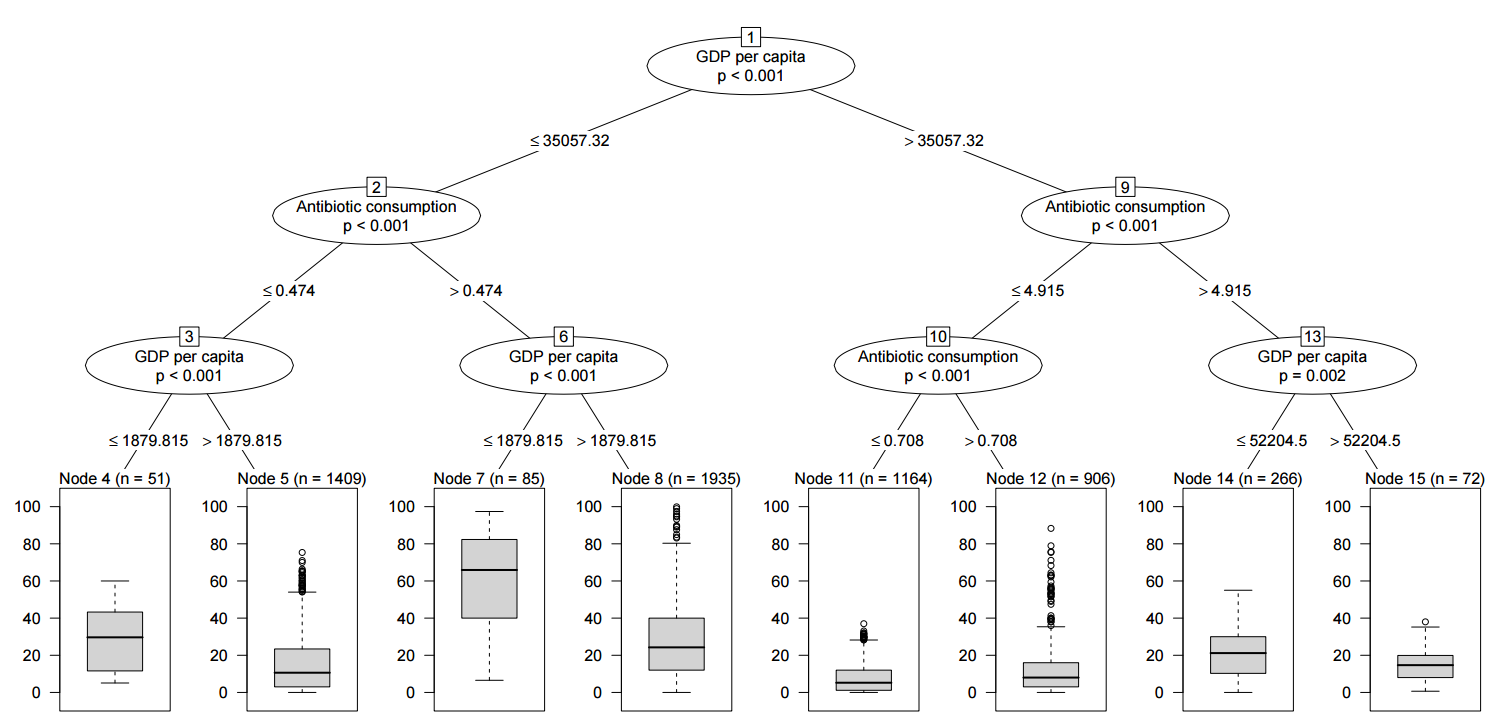


Note: The final estimates for this tree are the means of the box-plots at the bottom (e.g., a resistance proportion of around 70 percent for countries with a GPD per capita below or equal to $1,880 and antibiotic consumption above 0.474 standard units per 1,000 inhabitants)

There are a number of things to notice in the tree depicted above, not forgetting that the discussion that follows is simply to illustrate how regression trees work (i.e., no policy conclusions should be derived). First, countries with higher antibiotic consumption have higher predicted resistance proportions (see means on box-plots at the bottom of the figure; compare, for example, means of nodes 7 and 8 to those of nodes 4 and 5). However, it seems that the impact of antibiotic consumption on resistance proportions is mediated by GDP per capita (compare, for example, mean of node 8 to mean of node 4). More antibiotic consumption does not necessarily mean higher resistance proportions: per capita GDP must be taken into account. This exercise illustrates how difficult it can be to interpret a single, and simple, regression tree. A random forest is composed of thousands of regression trees, making it impossible to interpret its results without using simulations.

A key methodological development introduced by random forests is the reduction of correlation across trees. Unlike other tree-based methods (e.g., bagged trees), each branch of each tree in the random forest is constructed using a different subset *k* of all predictors *P* in the dataset. The value of the parameter *k*, commonly referred to as *m_try_* is a tuning parameter that is set to *P*/3 by default but can be optimised to minimise the RMSE [16]. In this study, the default *P*/3 was used. A random forest was fit to each of the 50 randomly selected imputed datasets, leading to 50 random forests, with 2,000 regression trees each. A third of the 150 estimates that informed each final estimate of the resistance proportion for a given country-antibiotic-bacterium-year combination were thus estimated using 100,000 regression trees.

While random forests are not easily interpretable, they have advantages over linear regression methods. First, all types of predictors (e.g., categorical and continuous) are handled naturally. Second, they are computationally easy to fit. Third, they require no formal distributional assumptions unlike, for example, linear regressions which assume normality. Fourth, they automatically fit non-linear interactions. Fifth, they automatically select the most important predictors (i.e., the predictors that maximise predictive power). These advantages justify the use of random forests as an alternative to linear regression.

### Incorporation of uncertainty

In line with concerns over previous estimations of future resistance proportions [1], we take into account uncertainty in the modelling process. There are many types of uncertainty that have an impact on the estimates discussed here. First, there is uncertainty in the underlying data. Differences in frequency of blood culture sampling, coverage and national representativeness of surveillance networks, as well as aggregation and reporting methods, all affect the quality of the data. Unfortunately, without more information on how these issues affect the resistance proportions reported in national and international surveillance networks, it is not possible to adjust the data to account for these potential sources of bias. A second type of uncertainty is captured by the algorithm Amelia II in the form of 150 imputed values for each missing observation. Capturing imputation uncertainty is especially important because of the number of missing values in the original dataset.

The third type of uncertainty is model selection. Uncertainty in what is the most appropriate model of AMR for each country-antibiotic-bacterium combination is incorporated in the use of three different classes of models with different assumptions and philosophies. This type of uncertainty is further captured by the use of different linear regression specifications for each country-antibiotic-bacterium combination. In the absence of strong evidence of which model is most appropriate, using three classes of models provides some relief from the implicit assumption that the relationships captured by each method in the past will persist in the future. Furthermore, the use of more than one method is supported by the assertion that combining forecasts from different sources leads, on average, to more accurate predictions than relying on a single source of forecasts [18]. Running each method on 50 randomly selected imputed sets propagates the uncertainty from the multiple imputations.

The fourth type of uncertainty is uncertainty in the model parameters. This type of uncertainty is captured by drawing new parameter estimates from a multivariate normal distribution of the estimated linear regression fixed effects (e.g. antibiotic consumption, real GDP, population over 64, and population under 15) as the mean and the model’s variance-covariance as the variance. Because the random forest is a stochastic process, a different seed is used each time to ensure that some uncertainty in the model’s parameters is also captured. Uncertainty in the parameters of the exponential smoothing method is not incorporated.

The fifth type of uncertainty is uncertainty in the forecasts of covariates. Unfortunately, the sources of forecasts of OOP health expenditure and real GDP do not report uncertainty intervals. While the UN WPP does report a confidence interval, incorporating it would require making a distributional assumption. For simplicity, uncertainty in the forecasts of covariates is not incorporated. Nevertheless, future antibiotic consumption is estimated for each of the 150 imputed datasets, thus propagating uncertainty in multiple imputations. The final estimates thus incorporate uncertainty in the imputations of missing values, in the model selection and specification, and in the models’ parameters.

The impact of uncertainty is visible in the following illustrative examples. As expected, countries for which more data points are available have narrower uncertainty intervals. However, uncertainty goes beyond data availability. The figures below illustrate a diversity in patterns across countries and antibiotic-bacterium combinations, again motivating the use of an ensemble of different classes of models.

**Figure 4. Resistance proportions for third-generation cephalosporin-resistant *E. coli* in Germany**


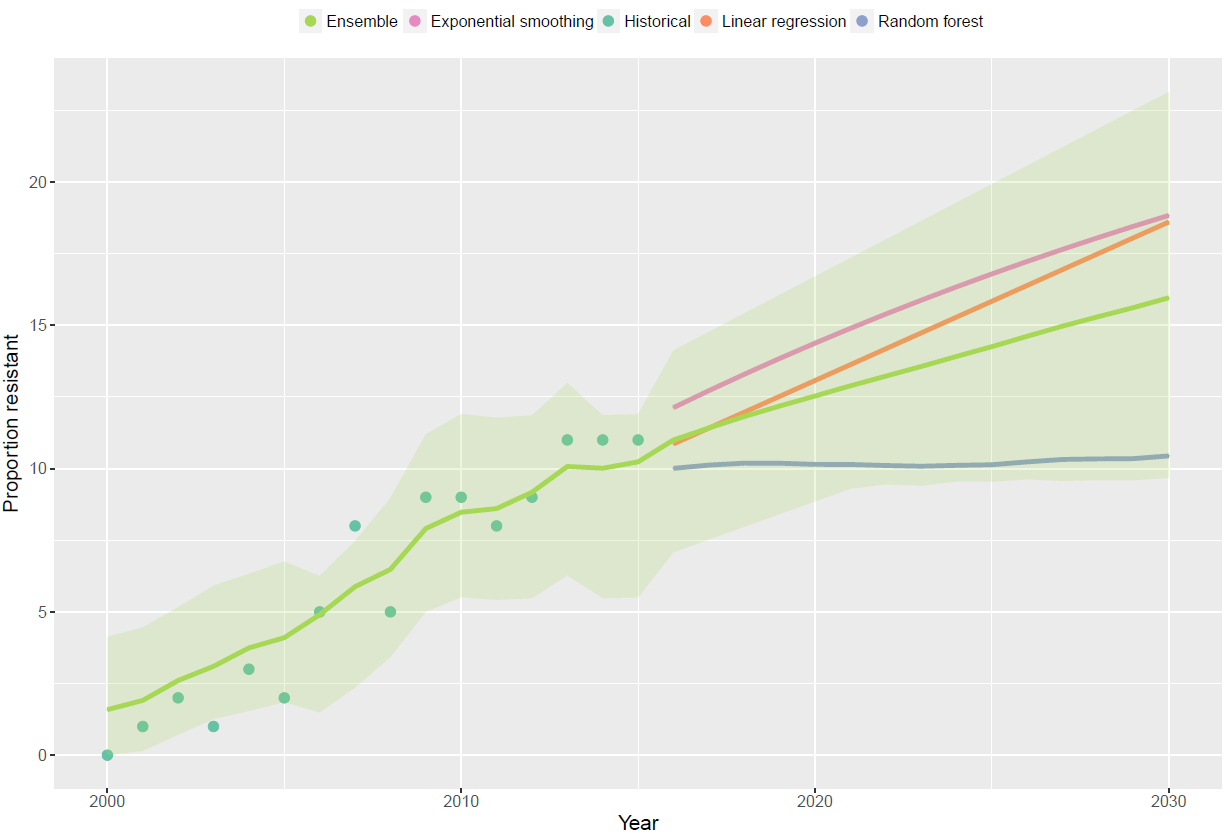


**Figure 5. Resistance proportions for third-generation cephalosporin-resistant *E. coli* in Hungary**


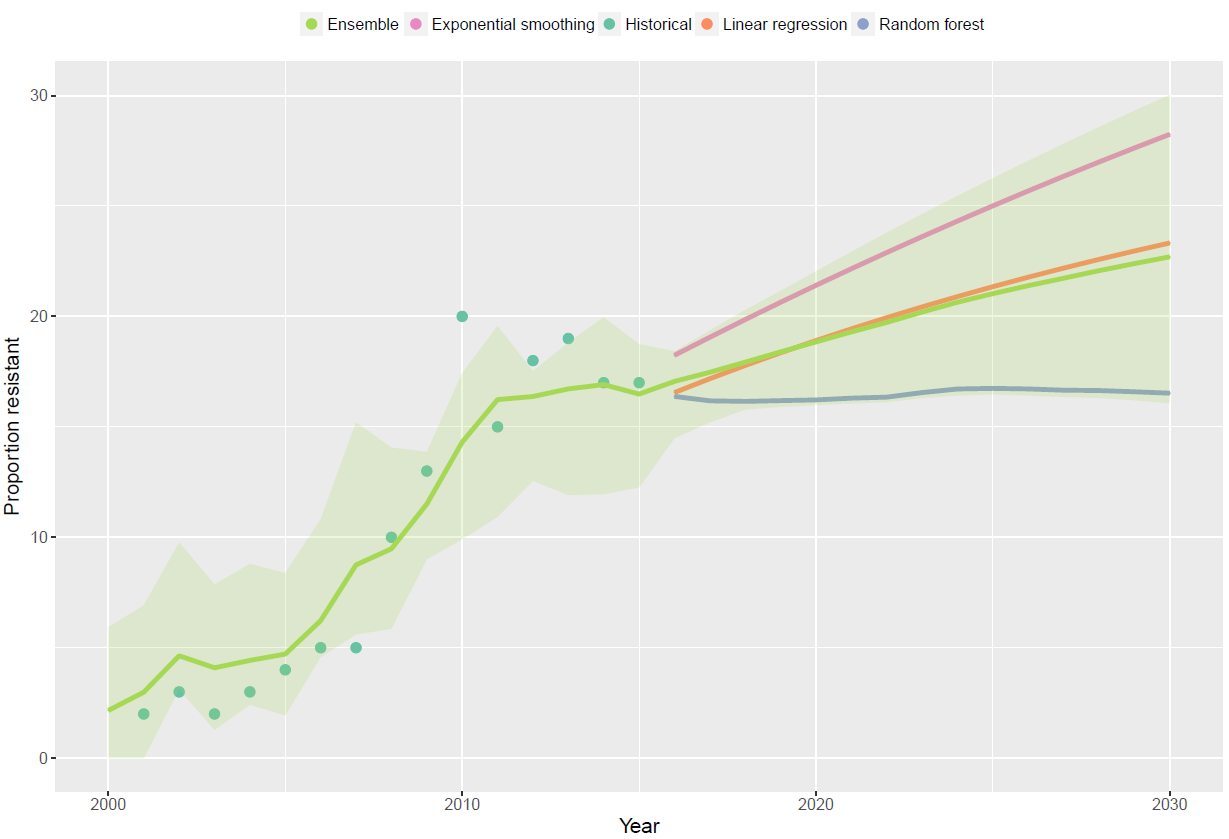


**Figure 6. Resistance proportions for penicillin-resistant *S. pneumoniae* in Sweden**


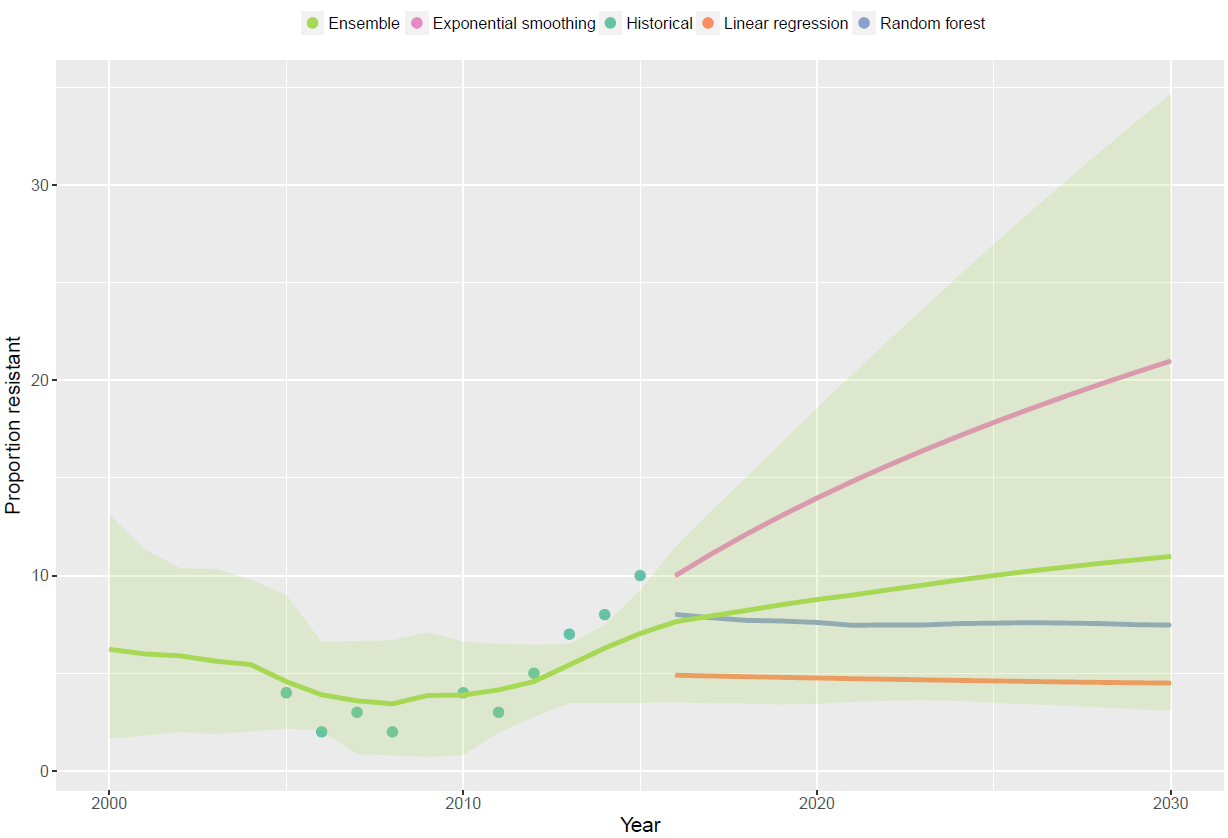


**Figure 7. Resistance proportions for penicillin-resistant *S. pneumoniae* in the United States**


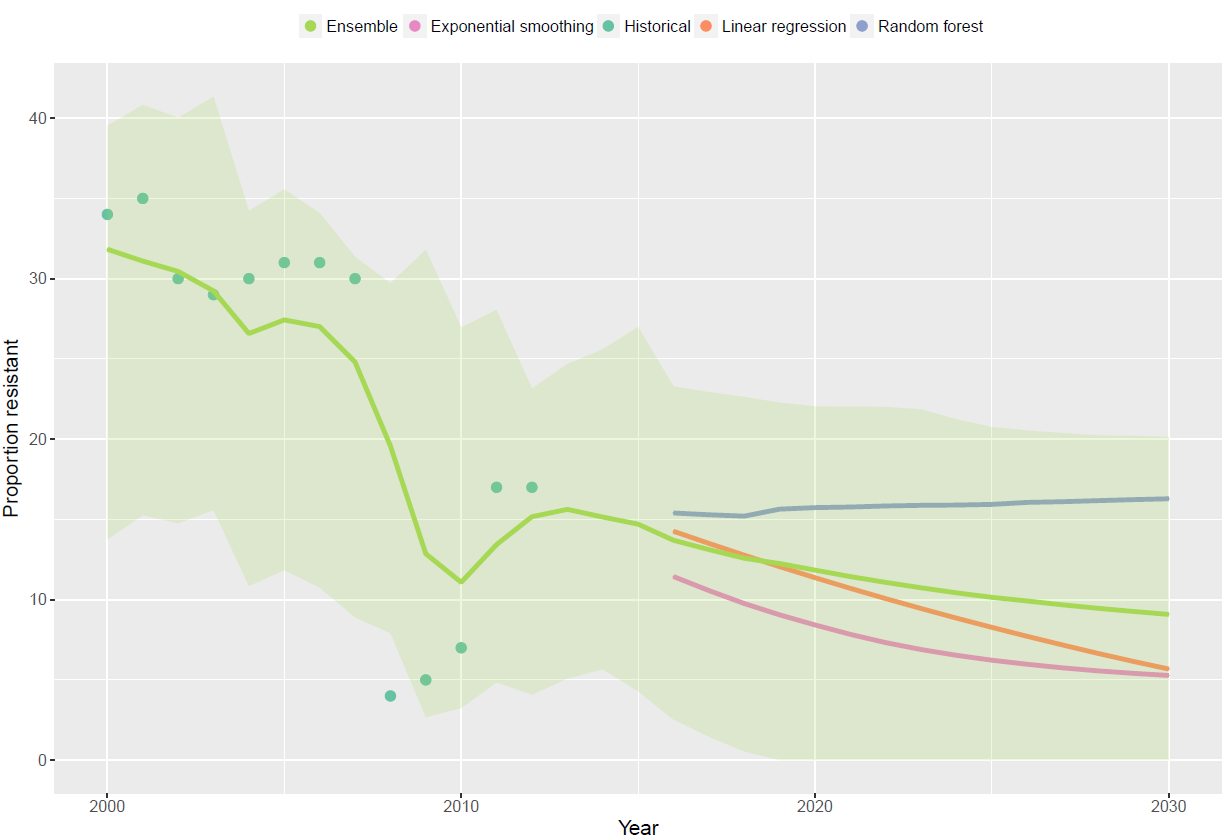


**Figure 8. Resistance proportions for MRSA in the Czech Republic**


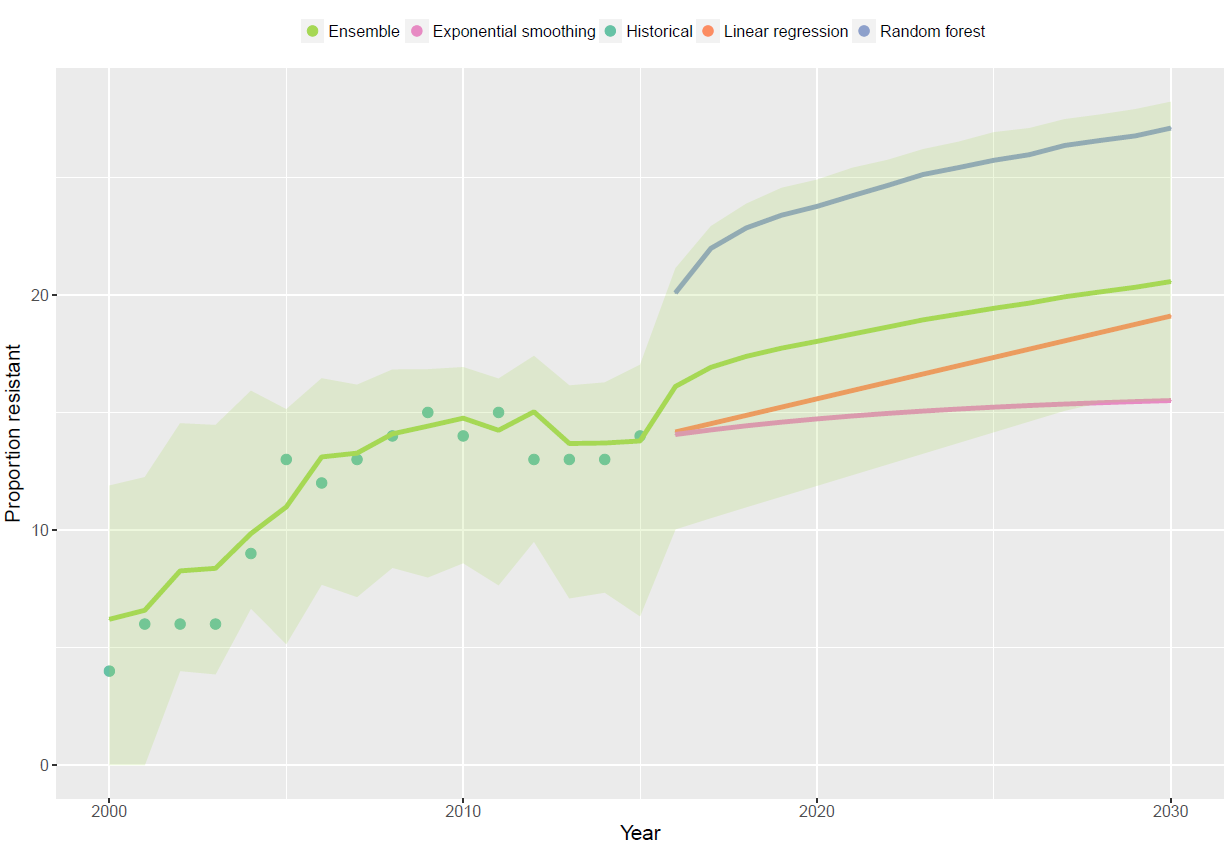


**Figure 9. Resistance proportions for MRSA in the Slovakia**


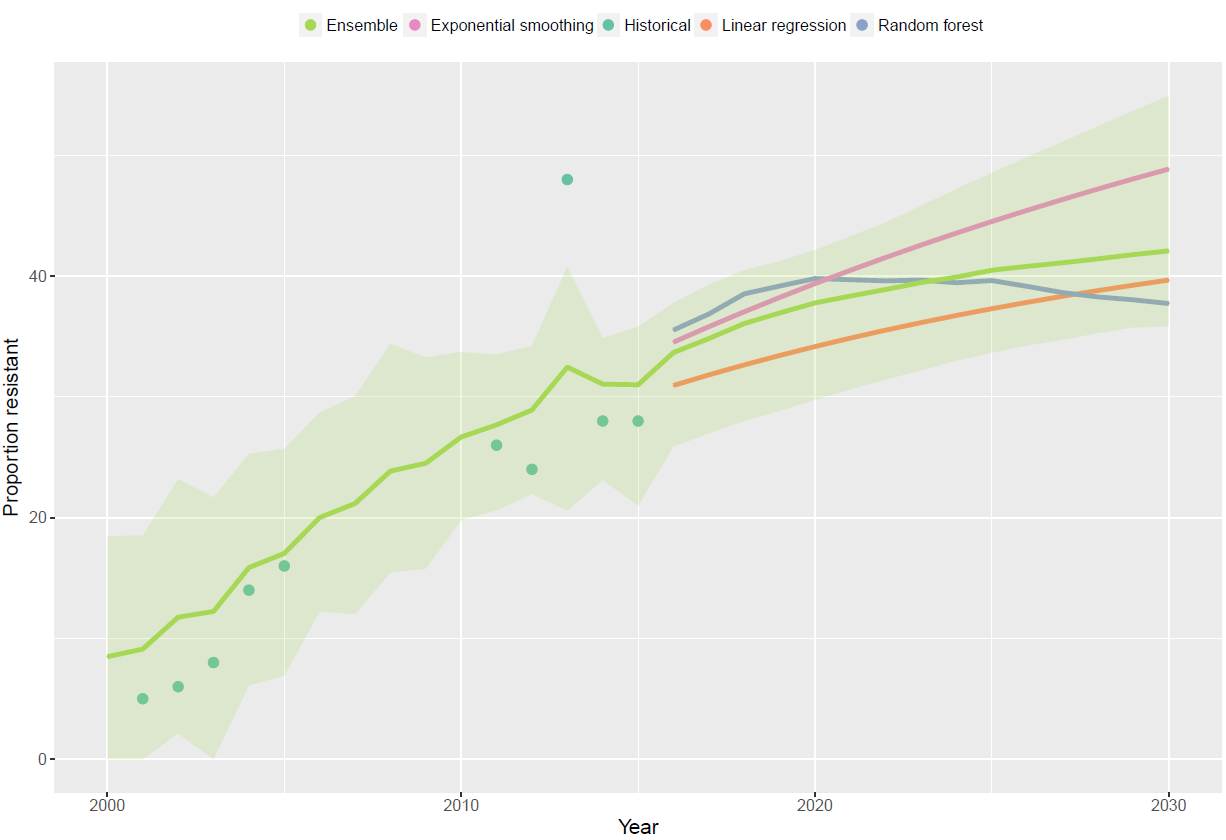


**Figure 10. Resistance proportions for carbapenem-resistant *P. aeruginosa* in Lithuania**


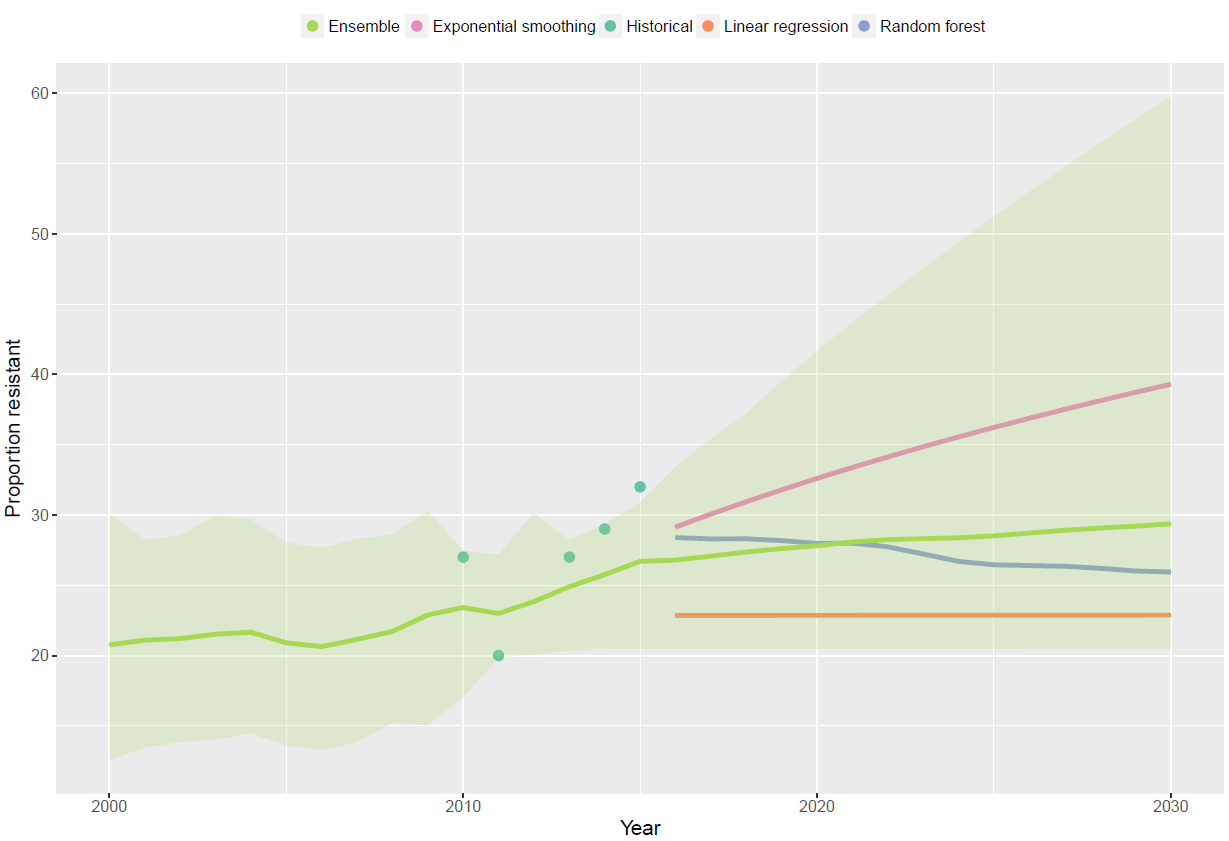


**Figure 11. Resistance proportions for carbapenem-resistant *P. aeruginosa* in South Africa**


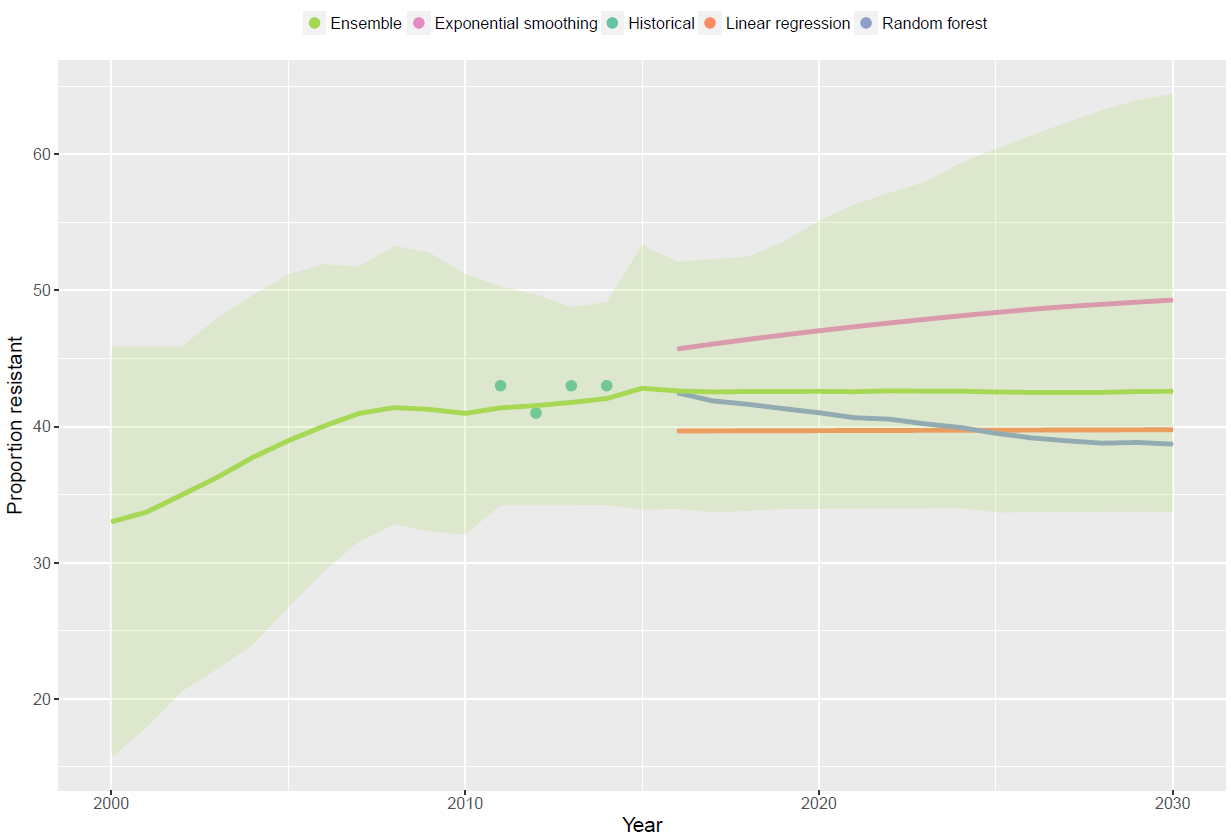


**Figure 12. Resistance proportions for 3rd-gen. cephalosporin-resistant *K. pneumoniae* in Italy**


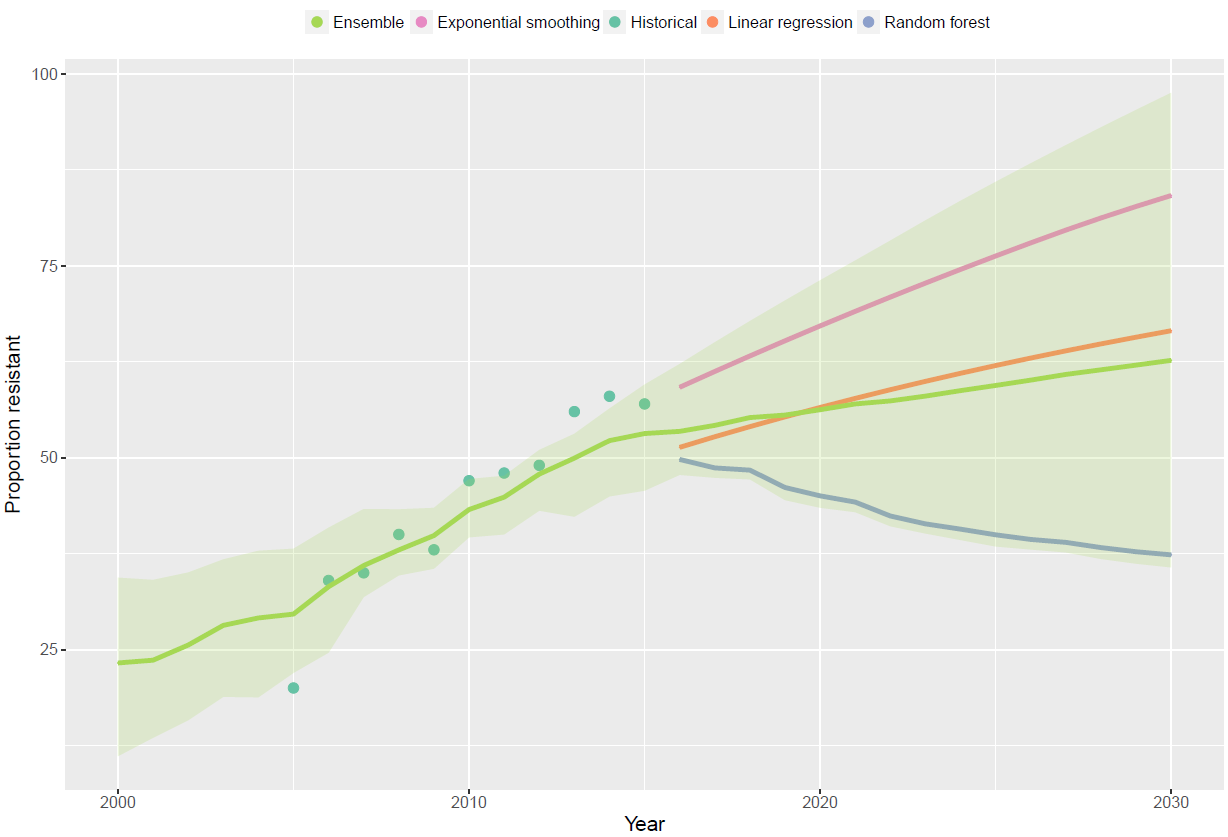


**Figure 13. Resistance proportions for 3rd-gen. cephalosporin-resistant *K. pneumoniae* in Spain**


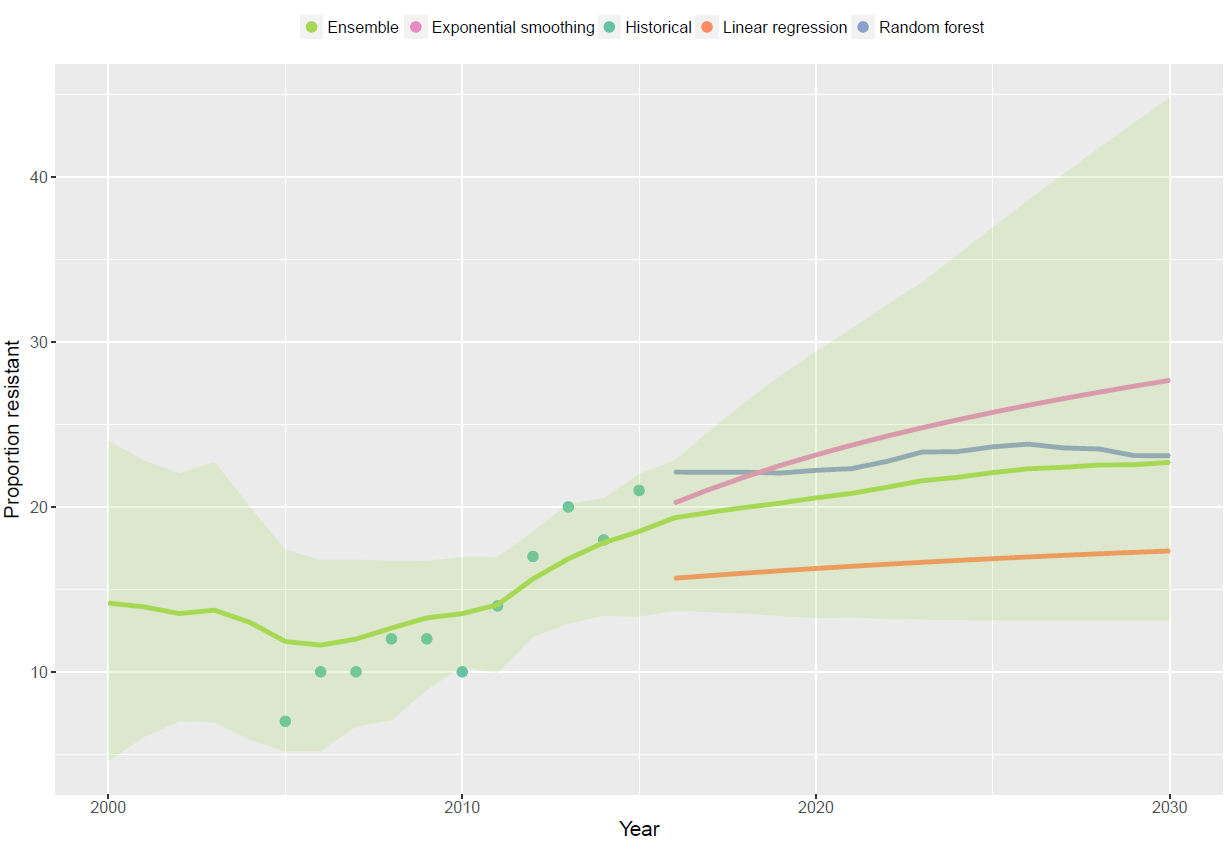


### Limitations of this study

There are important challenges in using a data-driven approach to forecast resistance proportions. The availability of comparable high-quality data on resistance proportions, and correlates of resistance, across a large number of countries is limited. Data on some indicators likely to be associated with resistance proportions – such as antibiotic consumption and resistance in the animal sector – could not be found, and so were not included. Despite efforts from ResistanceMap to harmonize the data (e.g. including only invasive isolates from blood and cerebrospinal fluid, categorizing results according to up-to-date CLSI/EUCAST criteria, etc.), differences in frequency of blood culture sampling, coverage and national representativeness of surveillance networks, as well as aggregation and reporting methods, should be considered when comparing proportions across countries. Information on past, current and future policies that might affect resistance proportions have not been explicitly accounted for (although country effects are used and should capture some of the between-country variation in resistance proportions). However, the statistical models described here can be readily updated as new and higher quality data become available (for example, through the WHO Global Antimicrobial Resistance Surveillance System or GLASS, or networks such as EARS-Net). This would lead to more accurate, reliable and informative estimates.

It is important to note the difference between prediction and interpretation. To paraphrase Kuhn and Johnson [16], the foremost objective of this approach is not to understand why resistance proportions will change in the future, but to accurately project the chances that they will change in the future. Unfortunately, achieving predictive accuracy typically comes at the cost of interpretability. To account for uncertainty in model selection and specification, this study combines the results of three classes of models. One of those methods, random forest, is difficult to interpret because, among other reasons, it aggregates the predictions of thousands of uncorrelated regression trees which allow explanatory variables to interact non-linearly. Interpreting one of the trees that make up a random forest would be challenging, interpreting the 2,000 trees that make up each of the 50 random forests that informed the estimates in this study is infeasible. Determining what is driving historical resistance proportions requires a different approach than the one employed here.

Any forecasting model which relies on past relationships to predict the future is not able to predict changes in those relationships. It is relatively easy to think of future changes that are not possible to account for in the models described here, from the more obvious ones (e.g. policies that limit, or otherwise, the use of antibiotics in the human and animal sectors) to the more unusual (e.g. climate change could lead to increased urbanisation, conflict and population displacement, all of which might affect resistance proportions). While the use of three classes of methods does limit interpretability, it should help minimize the impact of incorrectly projecting historical patterns into the future.

Finally, it is important to note this study estimates resistance proportions, not number of infections. The overall burden of infections from antimicrobial-resistant bacteria is a consequence of both the share of infections from resistant bacteria (estimated here) but also the number of new overall infections, or incidence (not estimated here). These two indicators can evolve differently over time.

### 9. References

1. de Kraker MEA, Stewardson AJ, Harbarth S. Will 10 Million People Die a Year due to Antimicrobial Resistance by 2050? PLOS Med [Internet]. 2016 Nov 29 [cited 2018 Jan 16];13(11):e1002184. Available from: http://dx.plos.org/10.1371/journal.pmed.1002184

2. KPMG LLP. The global economic impact of anti-microbial resistance [Internet]. 2014 [cited 2018 Jan 16]. Available from: https://home.kpmg.com/content/dam/kpmg/pdf/2014/12/amr-report-final.pdf

3. Center for Disease Dynamics E and P (CDDEP). ResistanceMap [Internet]. [cited 2018 Jul 24]. Available from: https://resistancemap.cddep.org/index.php

4. WHO. Antimicrobial resistance: global report on surveillance 2014. 2014. 257 p.

5. Australian Commission on Safety and Quality in Health Care (ACSQHC). AURA 2017: Second Australian report on antimicrobial use and resistance in human health [Internet]. 2017 [cited 2019 Feb 20]. Available from: http://www.safetyandquality.gov.au/antimicrobial-use-and-

6. Australian Commission on Safety and Quality in Health Care (ACSQHC). AURA 2016: first Australian report on antimicrobial use and resistance in human health [Internet]. 2016 [cited 2019 Feb 20]. Available from: www.safetyandquality.gov.au.

7. Byarugaba D. Antimicrobial resistance in developing countries and responsible risk factors. Int J Antimicrob Agents [Internet]. 2004 Aug 1 [cited 2018 Jul 24];24(2):105–10. Available from: https://www.sciencedirect.com/science/article/pii/S0924857904000895

8. Harbarth S, Samore MH. Antimicrobial resistance determinants and future control. Emerg Infect Dis [Internet]. 2005 Jun [cited 2018 Jul 24];11(6):794–801. Available from: http://www.ncbi.nlm.nih.gov/pubmed/15963271

9. Holmes AH, Moore LSP, Sundsfjord A, Steinbakk M, Regmi S, Karkey A, et al. Understanding the mechanisms and drivers of antimicrobial resistance. Lancet (London, England) [Internet]. 2016 Jan 9 [cited 2019 Feb 2];387(10014):176–87. Available from: http://www.ncbi.nlm.nih.gov/pubmed/26603922

10. Chatterjee A, Modarai M, Naylor NR, Boyd SE, Atun R, Barlow J, et al. Quantifying drivers of antibiotic resistance in humans: a systematic review. Lancet Infect Dis [Internet]. 2018 Dec 1 [cited 2019 Feb 2];18(12):e368–78. Available from: http://www.ncbi.nlm.nih.gov/pubmed/30172580

11. Liu JX, Goryakin Y, Maeda A, Bruckner T, Scheffler R. Global Health Workforce Labor Market Projections for 2030. Hum Resour Health [Internet]. 2017 Dec 3 [cited 2018 Jul 24];15(1):11. Available from: http://human-resources-health.biomedcentral.com/articles/10.1186/s12960-017-0187-2

12. United Nations Department of Economic and Social Affairs Population Division. World Population Prospects: The 2017 Revision, Volume I: Comprehensive Tables [Internet]. 2017. Available from: https://esa.un.org/unpd/wpp/Publications/Files/WPP2017_Volume-I_Comprehensive-Tables.pdf

13. United States Department of Agriculture (USDA) Economic Research Service. International Macroeconomic Data Set [Internet]. 2017 [cited 2018 Jul 24]. Available from: https://www.ers.usda.gov/data-products/international-macroeconomic-data-set/

14. Global Burden of Disease Health Financing Collaborator Network JL, Campbell M, Chapin A, Eldrenkamp E, Fan VY, Haakenstad A, et al. Future and potential spending on health 2015-40: development assistance for health, and government, prepaid private, and out-of-pocket health spending in 184 countries. Lancet (London, England) [Internet]. 2017 May 20 [cited 2018 Jul 24];389(10083):2005–30. Available from: http://www.ncbi.nlm.nih.gov/pubmed/28433260

15. Honaker J, King G, Blackwell M. Amelia II: A Program for Missing Data. J Stat Softw [Internet]. 2011 Dec 12 [cited 2018 Jul 24];45(7):1–47. Available from: http://www.jstatsoft.org/v45/i07/

16. Kuhn M, Johnson K. Applied Predictive Modeling [Internet]. New York: Springer; 2016 [cited 2018 Jul 24]. Available from: https://link.springer.com/content/pdf/10.1007/978-1-4614-6849-3.pdf

17. Hyndman RJ, Athanasopoulos G. Forecasting : principles and practice. OTexts; 2018.

18. Tetlock P, Gardner D. Superforecasting: The art and science of prediction. New York: Broadway Books; 2016.
